# Building-Scale Wastewater Metagenomics Reveals Temporal Patterns in Resistance and Virulence Genes

**DOI:** 10.3390/antibiotics15090878

**Published:** 2026-09-08

**Authors:** Ugonna C. Morikwe, Larisa C. Kiki, Franklin C. Ezeanowai, Shamiah Hall, Shilpi Bhatia, Tinyiko Nicole Maswanganye, Olusola Jeje, Megan S. Hill, Joseph L. Graves, Dongyang Deng, Liesl Jeffers-Francis

**Affiliations:** 1 Biology Department, College of Science and Technology, North Carolina A&T State University, 1601 E. Market Street, Greensboro, NC 27411, USA; ucmorikwe@aggies.ncat.edu (U.C.M.); klchila@aggies.ncat.edu (L.C.K.); cfezeanowai@aggies.ncat.edu (F.C.E.); snhall@aggies.ncat.edu (S.H.); sbhatia@ncat.edu (S.B.); nicolemaswanganye@ymail.com (T.N.M.); ojeje@ncat.edu (O.J.); gravesjl@ncat.edu (J.L.G.J.); 2 Department of Civil & Environmental Engineering, PRATT School of Engineering, Duke University, Durham, NC 27708, USA; megan.hill@duke.edu; 3 Department of Built Environment/Environmental Health and Safety Program, College of Science and Technology, North Carolina A&T State University, 1601 E. Market Street, Greensboro, NC 27411, USA; ddeng@ncat.edu

**Keywords:** wastewater-based epidemiology, antibiotic resistance genes, virulence factors, shotgun metagenomics, resistome and virulome, sewershed surveillance, co-occurrence network, COVID, microbiome, built environment

## Abstract

**Background/Objectives:** Antimicrobial resistance (AMR) and virulence represent co-evolving dimensions of microbial pathogenic potential whose ecological organization in building-scale wastewater systems remains poorly understood. **Methods:** Using shotgun metagenomic sequencing, we characterized the temporal dynamics and ecological associations of antimicrobial resistance genes (ARGs) and virulence factors (VFs) in 12 wastewater grab samples (2 per semester) collected from a university residence hall designated for COVID-19 quarantine between 2021 and 2023. **Results:** The wastewater microbiome was anchored by a stable core of gut-associated anaerobic bacteria, with community composition exhibiting significant Spring-versus-Fall structuring and a year × semester interaction that explained 60% of the community variation. A marked shift toward opportunistic taxa, particularly *Acinetobacter*, during Fall 2023 represented the most pronounced temporal perturbation. Total ARG abundance remained stable across semesters, while resistome composition shifted significantly, indicating that temporal dynamics were driven by compositional turnover rather than changes in overall resistance burden. VF functional categories were broadly conserved across sampling periods, consistent with their structural embedding within the persistent fecal core microbiome. Correlation and network analyses revealed modular ecological coupling between resistance and virulence functional categories, with metal/co-resistance and fosfomycin classes showing the strongest associations with virulence functions. At the community level, a Benjamini–Hochberg–corrected co-occurrence network resolved into taxa-anchored resistance modules and separate virulence-function clusters, with *Acinetobacter* and fluoroquinolone resistance as the principal connectors. **Conclusions:** These findings indicate that building-scale wastewater metagenomics can capture ecologically structured functional gene dynamics, highlighting its potential as a surveillance tool for monitoring AMR and virulence in built environments.

## 1. Introduction

Wastewater represents an integrative matrix of human and environmental microbial activity, reflecting the collective biological outputs of populations and the built environments they inhabit. As a composite of human-associated microbiota, environmental microbes, and chemical inputs, wastewater provides a systems-level view of microbial ecology shaped by host physiology, antimicrobial exposure, and environmental processes. Advances in wastewater-based epidemiology have demonstrated its utility for monitoring public health signals, including pathogen circulation, microbial community composition, and functional gene dynamics [1]. More recently, shotgun metagenomic sequencing has expanded these capabilities by enabling simultaneous characterization of microbial taxa and functional gene repertoires, including antimicrobial resistance genes (ARGs) and virulence factors (VFs), thereby transforming wastewater surveillance from targeted detection to comprehensive ecological profiling [2,3]. These developments position wastewater not only as a surveillance tool but also as a reservoir of functionally relevant microbial traits with implications for public health.

Among these, antimicrobial resistance (AMR) has emerged as a critical global concern, with wastewater systems recognized as important reservoirs and transmission pathways for ARGs. Metagenomic studies of municipal and environmental wastewater systems have consistently identified diverse and abundant ARGs across multiple resistance classes, highlighting the role of wastewater in disseminating AMR in the environment [2,4]. However, most investigations have focused on ARG occurrence and abundance at large spatial scales, with comparatively limited attention to the ecological organization of resistance genes within microbial communities, particularly in confined or fine-scale systems where population signals are more localized. In parallel, VFs represent key determinants of microbial pathogenic potential, enabling host colonization, persistence, and infection through mechanisms such as adhesion, immune evasion, and toxin production. Increasing evidence suggests that ARGs and VFs may co-occur within microbial genomes or mobile genetic elements, facilitating their joint dissemination under shared selective pressures [5,6]. This co-occurrence has important implications for public health, as it may enhance the pathogenic potential of resistant organisms. Wastewater environments, characterized by high microbial diversity, dense cell populations, and abundant mobile genetic elements, provide favorable conditions for horizontal gene transfer and functional gene exchange. Despite the growing number of studies profiling both resistomes and virulomes in wastewater, the few that jointly resolve taxa, ARGs, and VFs have largely done so at municipal or hospital scales [7,8], and comprehensive assessments of how resistance and virulence traits are ecologically coupled within microbial communities and at fine spatial resolutions remain limited [9,10].

This knowledge gap is particularly pronounced at the building level, where microbial inputs reflect more defined and temporally coherent human populations. In contrast to municipal systems, which aggregate signals from large, heterogeneous populations, building-scale wastewater provides greater sensitivity for detecting localized ecological dynamics and short-term perturbations in microbial community structure and function [9]. Such systems are especially informative in settings characterized by altered human behavior and antimicrobial usage, including quarantine or isolation environments during infectious disease outbreaks. During the COVID-19 pandemic, quarantine settings were characterized by altered occupancy patterns, elevated infection rates, intensified use of disinfectants, and disruptions to antimicrobial stewardship practices, which may influence both microbial community structure and functional gene dynamics [11,12]. However, few studies have examined how resistance and virulence traits are organized within microbial communities at this scale, and how their ecological relationships vary over time. How such wastewater-derived signals correspond to clinically observed resistance patterns remains an open question. Although wastewater-based studies have documented the presence and diversity of ARGs [4], the extent to which these profiles reflect clinically relevant resistance dynamics remains insufficiently resolved, particularly in localized systems.

In this study, we use metagenomic sequencing to investigate the temporal dynamics and ecological associations of microbial communities, ARGs, and VFs in wastewater collected from a university residence hall designated for COVID-19 quarantine between 2021 and 2023. We characterized microbial taxonomic composition, resistome and virulome profiles, and evaluated co-occurrence patterns and network structure to assess the ecological coupling of resistance and virulence traits. In addition, we contextualized these ecological patterns against campus-level COVID-19 testing and quarantine records from the university student health center to characterize the dynamics of occupancy and infection pressure during the study period. By integrating ecological analysis with this population-level context at a fine spatial scale, this work aims to elucidate the organization of functionally relevant genes in human-associated microbial communities and to advance the application of wastewater metagenomics for high-resolution public health surveillance.

## 2. Results

### 2.1. Gut-Associated Anaerobic Bacteria Dominate the Wastewater Microbiome

**Figure 1:**
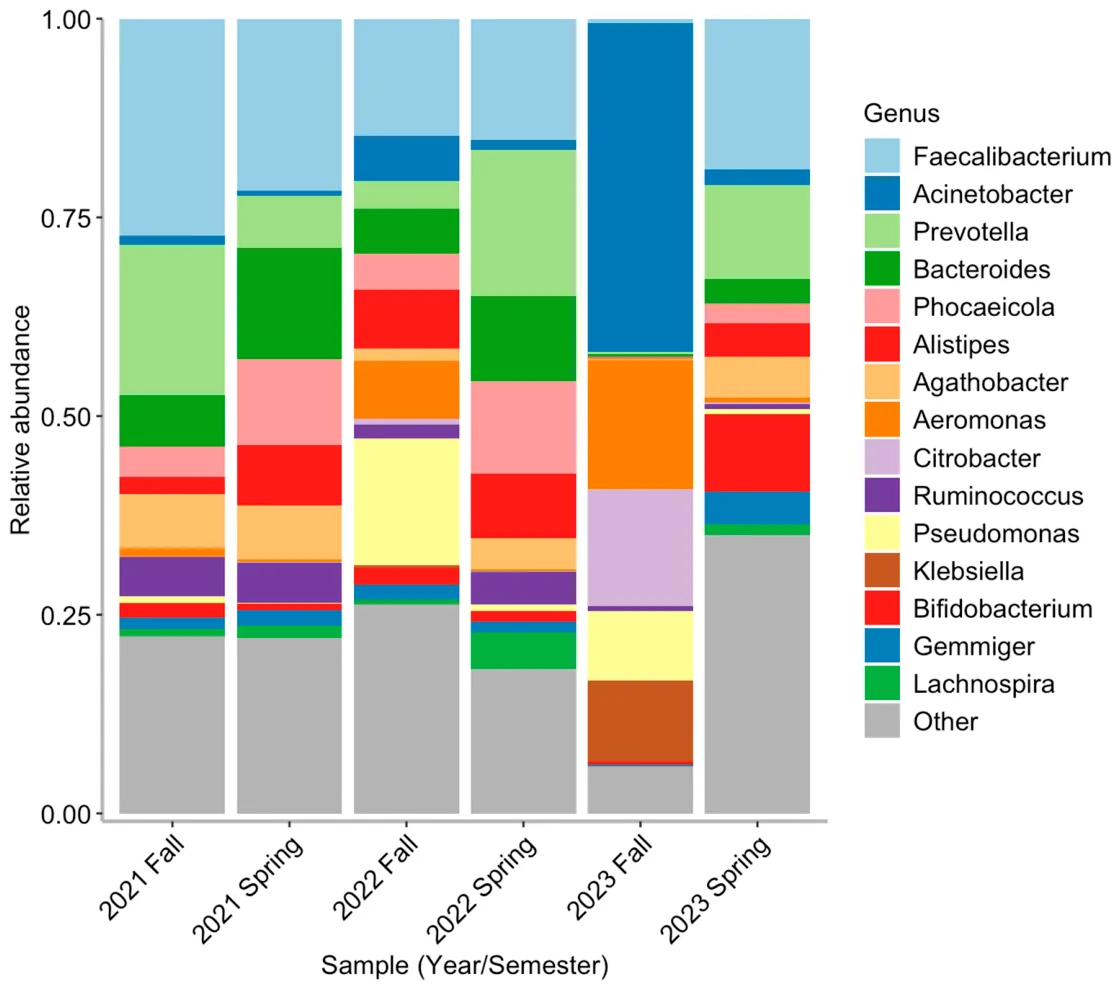
Genus-level microbiome composition across semesters.

Microbial community composition at the genus level was broadly consistent across sampling periods, with wastewater communities dominated by gut-associated bacterial taxa (Figure 1). Across samples, *Faecalibacterium* was the most abundant genus, accounting for a mean of 16.4% (range: 0.6–27.3%) of the total relative abundance, followed by *Prevotella* (mean: 10.0%, range: 0.4–19.0%), *Bacteroides* (mean: 6.7%, range: 0.2–14.1%), *Phocaeicola* (mean: 5.6%, range: 0.1–11.7%), and *Alistipes* (mean: 4.9%, range: 0.1–8.1%). Collectively, these five gut-associated genera accounted for a mean of 43.6% (range: 1.5–64.2%) of the total percent relative abundance across samples, indicating a dominant human fecal signature in the residence hall wastewater. Spring semester samples showed a higher mean combined contribution from these genera (mean: 54.1%, range: 35.8–69.6%) compared with Fall semester samples (mean: 29.0%, range: 1.5–60.0%), though this difference did not reach statistical significance (Wilcoxon rank-sum test, W = 27, *p* = 0.180), likely reflecting the limited sample size (*n* = 12). Despite this overall consistency, temporal variation in relative abundance was observed across semesters and years. Samples collected during Fall 2023 had a greater abundance of *Acinetobacter* compared with earlier sampling periods, whereas Spring 2023 samples showed higher relative contributions from gut-associated anaerobic genera (Figure 1). Several additional genera, including *Aeromonas*, *Citrobacter*, *Klebsiella*, *Pseudomonas*, and *Bifidobacterium*, were detected intermittently across samples, contributing to overall community composition. At the species level, the total abundance was concentrated in a small number of dominant taxa across samples (Figure S1).

### 2.2. Seasonal Patterns in Microbial Alpha and Beta Diversity

**Figure 2:**
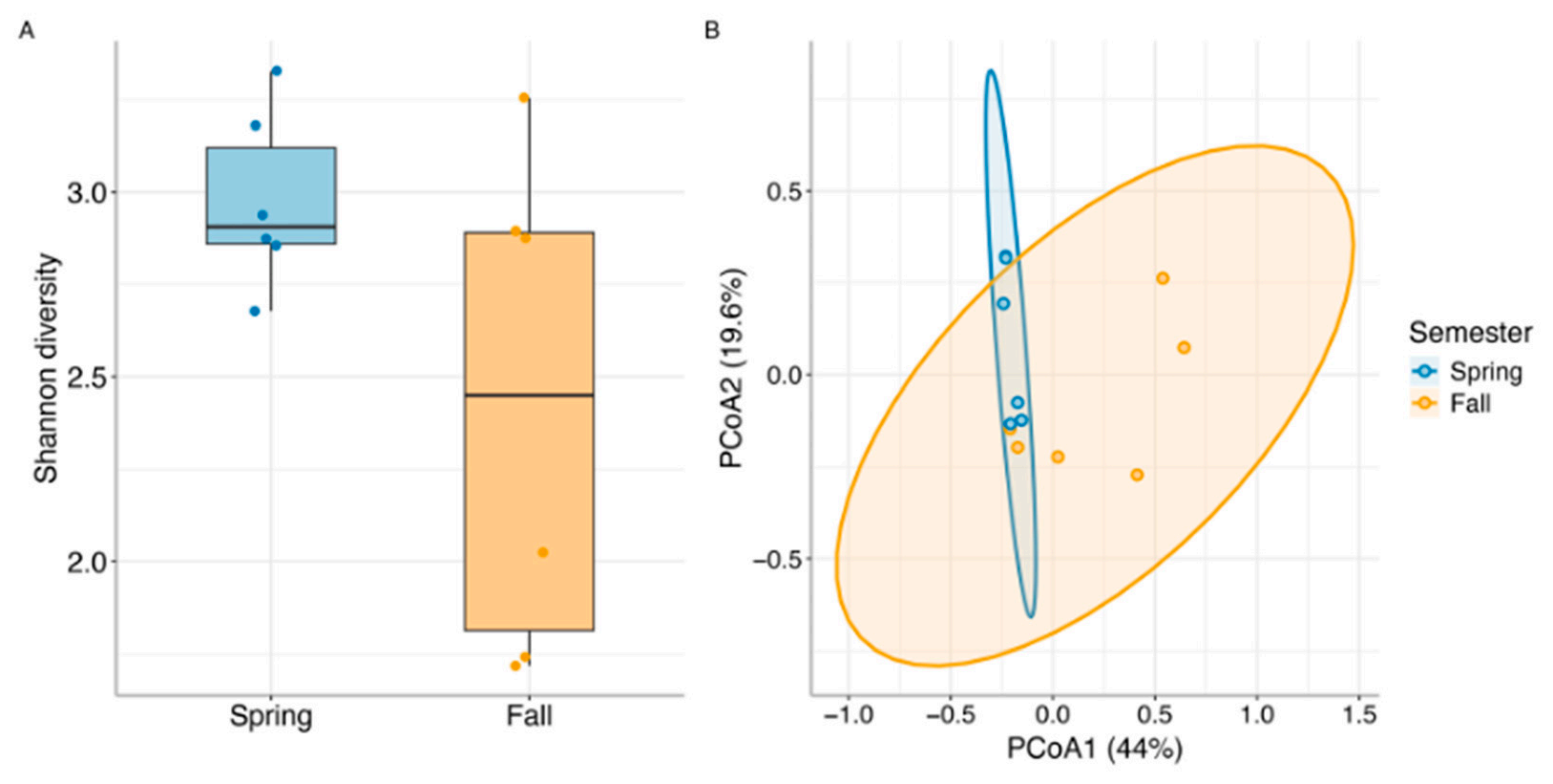
(**A**) Shannon diversity grouped by semester (Spring and Fall). (**B**) Bray–Curtis ordination by semester. Shannon diversity did not differ significantly between Spring and Fall (Wilcoxon rank-sum test, *p* > 0.05), whereas community composition differed significantly between semesters (PERMANOVA on Bray–Curtis distances, R^2^ = 0.23, *p* = 0.01).

Alpha diversity, measured by the Shannon index, did not differ significantly between semesters (Wilcoxon rank-sum test, *p* > 0.05; Figure 2A). Spring samples exhibited higher and more consistent diversity values, whereas Fall samples showed lower median and greater variability among samples. This was reflected in species richness values, with an average of 109 genera in the Spring semesters and 84.2 in the Fall (*p* = 0.31), and greater variability in the Fall (range: 43–124) than in the Spring (range: 92–125). Patterns of between-sample community structure were assessed using principal coordinates analysis (PCoA) based on Bray–Curtis dissimilarity (Figure 2B). Samples clustered by semester along the first principal coordinate, which accounted for 44% of the total variation, indicating distinct community compositions between the Spring and Fall sampling periods (PERMANOVA, R^2^ = 0.23, *p* = 0.01). However, dispersion also differed significantly between groups (PERMDISP, *p* = 0.045), reflecting differences in both community composition and within-group variability. Spring samples formed a relatively tight cluster, whereas Fall samples showed greater dispersion in ordination space, reflecting greater heterogeneity in community composition. While year alone was not significant (R^2^ = 0.16, *p* = 0.076), the combined model including year and semester was statistically significant (R^2^ = 0.60, *p* = 0.001), indicating that seasonal patterns varied across years.

### 2.3. Total Antimicrobial Resistance Genes Abundance and Class-Level Resistome Across Semesters

**Figure 3:**
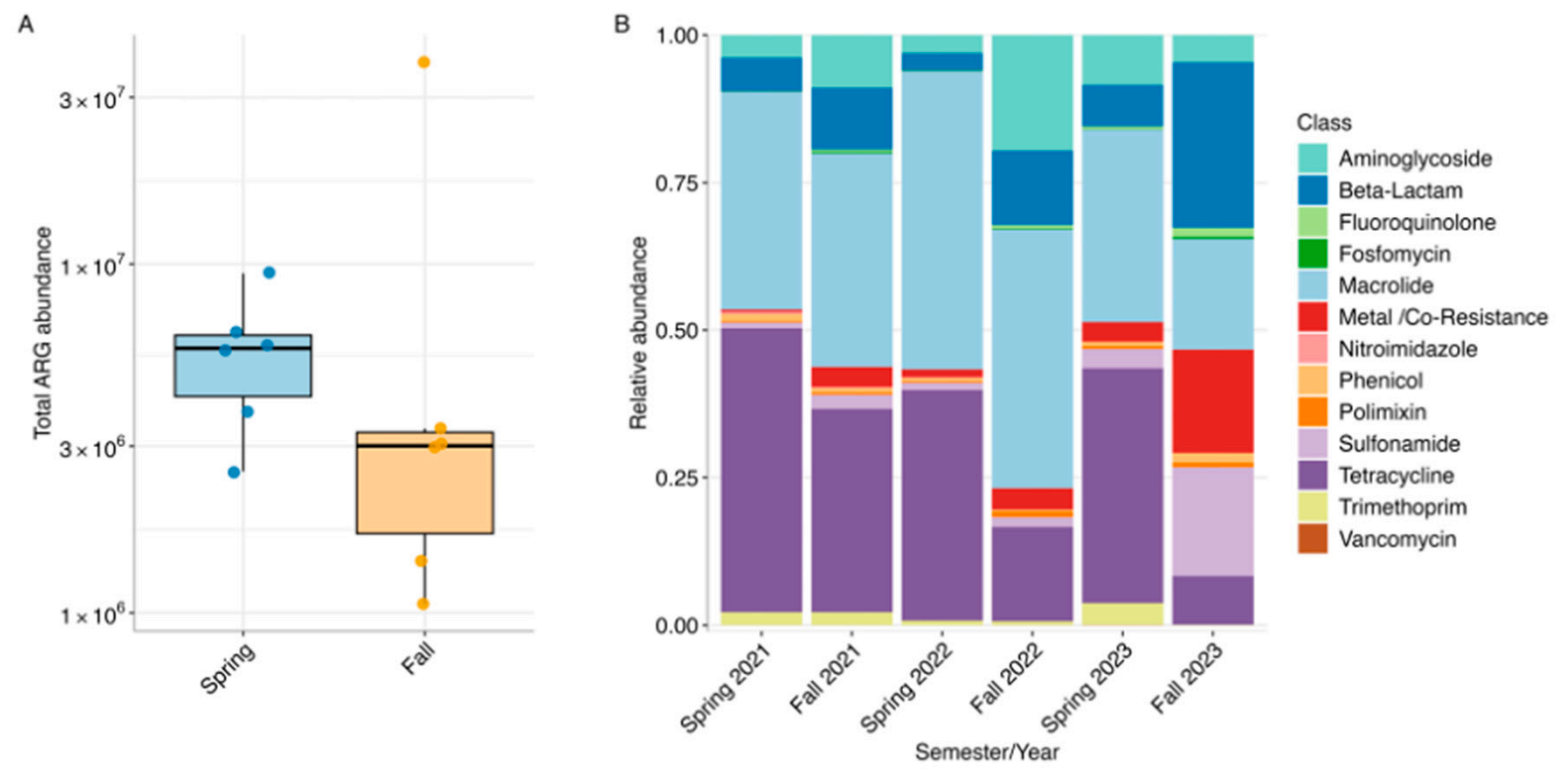
(**A**) Total antimicrobial resistance gene (ARG) abundance by semester (Spring vs. Fall). (**B**) Relative abundance of class-level resistome categories by Semester-year (2021–2023).

Total ARG abundance did not differ significantly between semesters (Wilcoxon rank-sum, *p* > 0.05; Figure 3A), and there was no monotonic temporal trend over the study period (Spearman ρ = 0.03, *p* = 0.927). Spring samples showed higher median total ARG abundance (5.73 × 10^6^) than Fall samples (3.01 × 10^6^), whereas Fall exhibited greater variability (SD = 1.45 × 10^7^), largely driven by an elevated ARG abundance in Fall 2023 (mean = 2.05 × 10^7^). Consistent with these patterns, a Kruskal–Wallis test across all semester-year groups detected no statistically significant temporal differences in total ARG abundance (χ^2^ = 7.08, df = 5, *p* = 0.215). ARG α-diversity likewise showed no strong evidence of temporal structuring, with non-significant correlations for richness (Spearman ρ = 0.49, *p* = 0.106) and Shannon diversity (ρ = 0.24, *p* = 0.459), and no significant Spring–Fall contrasts (Wilcoxon, *p* > 0.05). Taken together, these results indicate fluctuations in total ARG abundance and diversity over time rather than a clear directional shift within the 3-year sampling window.

Resistome composition by ARG class was broadly similar across semesters and years (Figure 3B). Macrolide and tetracycline resistance dominated across all periods (mean relative abundances of 36.5% and 30.9%, respectively), whereas the sulfonamide, polymyxin, and vancomycin classes contributed smaller fractions. Although the relative contributions of several classes differed across semester–year combinations, omnibus Kruskal–Wallis tests remained non-significant after Benjamini–Hochberg correction for false discovery rate (FDR) (all adjusted *p* > 0.05; raw *p*-values for individual class comparisons are reported in Table S1). Beta-lactam genes showed higher median relative abundance in Fall (14.9%) than in Spring (5.3%), while tetracycline displayed the opposite pattern (Spring median = 42.2% vs. Fall median = 15.9%); metal/co-resistance genes also had higher Fall median abundance (4.4%) compared to Spring (1.3%). Nitroimidazole resistance was the only class exhibiting a statistically significant directional decrease over time (Spearman ρ = −0.791, adjusted *p* = 0.028, Figure S2). Fall 2023 was particularly notable for elevated relative abundances of beta-lactam (28.0%), sulfonamide (18.4%), and metal/co-resistance genes (17.5%) with fold changes of 3.6–9.5 compared to other periods; these differences were not statistically significant after FDR correction (all adjusted *p* > 0.05).

Beta-lactam resistance genes were further examined at the subtype level (Figure S3), given their pronounced interannual variability (CV = 84.8%), significant seasonal enrichment in Fall semesters, and marked compositional shifts observed in Fall 2023. Serine β-lactamases accounted for about 99.9% of total β-lactam ARG abundance, with Class A (mean = 42.7 ± 25.2%) and Class D enzymes (mean = 39.3 ± 15.3%) predominating, followed by Class C (17.9 ± 13.0%) and Class B metallo-β-lactamases (<1%). Within Class A, carbapenemases were primarily driven by *bla*KPC, approximately 4–5-fold more abundant than individual ESBL genes (*bla*TEM, *bla*PER, *bla*VEB), with carbapenemases collectively predominating within Class A. Subtype analysis revealed extensive diversification with 40 variants in Class C and 38 in Class D β-lactamases. Notably, Class C AmpC-type genes *bla*CMY and *bla*MOX showed significantly higher abundance in Fall compared to Spring semesters (Wilcoxon rank-sum, adjusted *p* = 0.017 for both), and *bla*CMY additionally exhibited a significant increasing temporal trend across the study period (Spearman ρ = 0.307, adjusted *p* < 0.001), suggesting progressive enrichment of this AmpC-type cephalosporinase independent of seasonal variation. Class D resistance was dominated exclusively by *bla*OXA-type genes across all sampling periods. Aminoglycoside resistance comprised seven subclasses, with *aadA* nucleotidyltransferase genes representing the largest proportion of total abundance (2.13 × 10^6^), followed by *strB* and *strA* phosphotransferases (Figure S4); no significant seasonal differences were observed after FDR correction (all *p* > 0.05).

**Figure 4:**
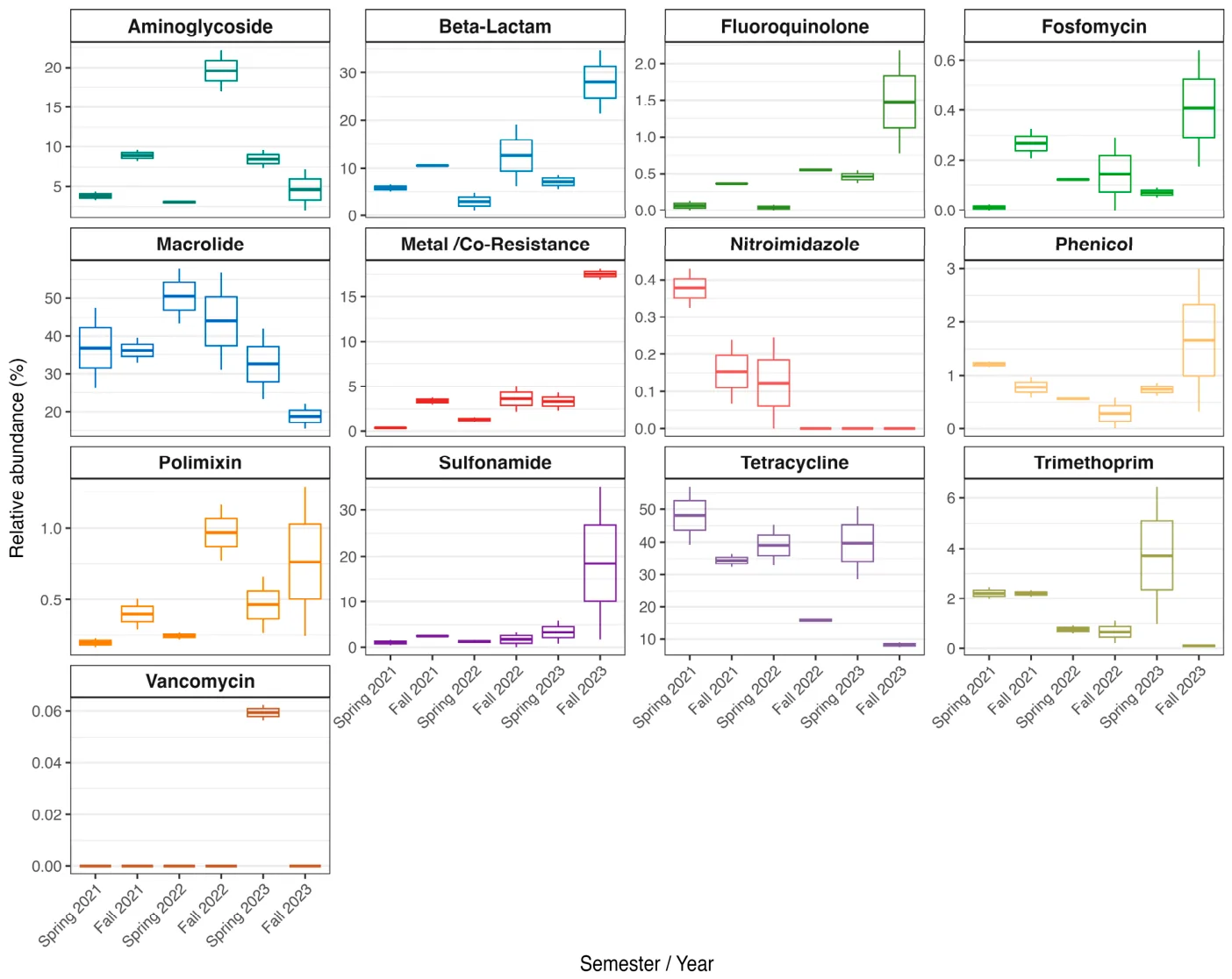
Distribution of ARG class relative abundance across semesters (2021–2023). Boxplots show the variation in relative abundance (%) of ARG classes detected in wastewater samples collected from NC A&T quarantine dormitory (Haley Hall) across Spring and Fall semesters from 2021 to 2023. Each panel represents an ARG class, with the median, interquartile range, and outliers indicated.

**Figure 5:**
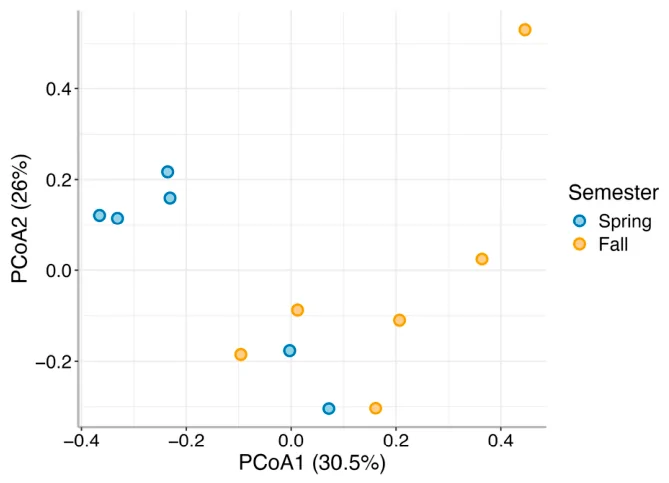
PCoA of gene-level ARG profiles (Bray–Curtis). Resistome composition differed significantly between semesters (PERMANOVA, R^2^ = 0.23, *p* = 0.001).

The distribution of relative ARG class abundances was further assessed across semester-year periods (Figure 4). Beta-lactam resistance showed higher median relative abundance in Fall periods (median = 14.9%) compared to Spring (median = 5.3%; Wilcoxon, adjusted *p* = 0.118; Table S1), while nitroimidazole, vancomycin, and fosfomycin remained consistently low across most sampling periods (mean < 0.2%). Collectively, these patterns indicate that although overall resistome composition remained stable at the class level, the magnitude and dispersion of individual resistance classes varied across years. Principal coordinates analysis (PCoA) based on Bray–Curtis dissimilarities revealed separation of ARG profiles by semester (Figure 5). PERMANOVA indicated that resistome composition differed significantly between Spring and Fall samples (R^2^ = 0.23, F = 2.97, *p* = 0.001), with semester accounting for 23% of the variation in gene-level ARG structure. Homogeneity of multivariate dispersion was supported (PERMDISP, *p* = 0.24), reinforcing the validity of the PERMANOVA result.

### 2.4. Virulence Factor Abundance and Functional Composition Across Semesters

**Figure 6:**
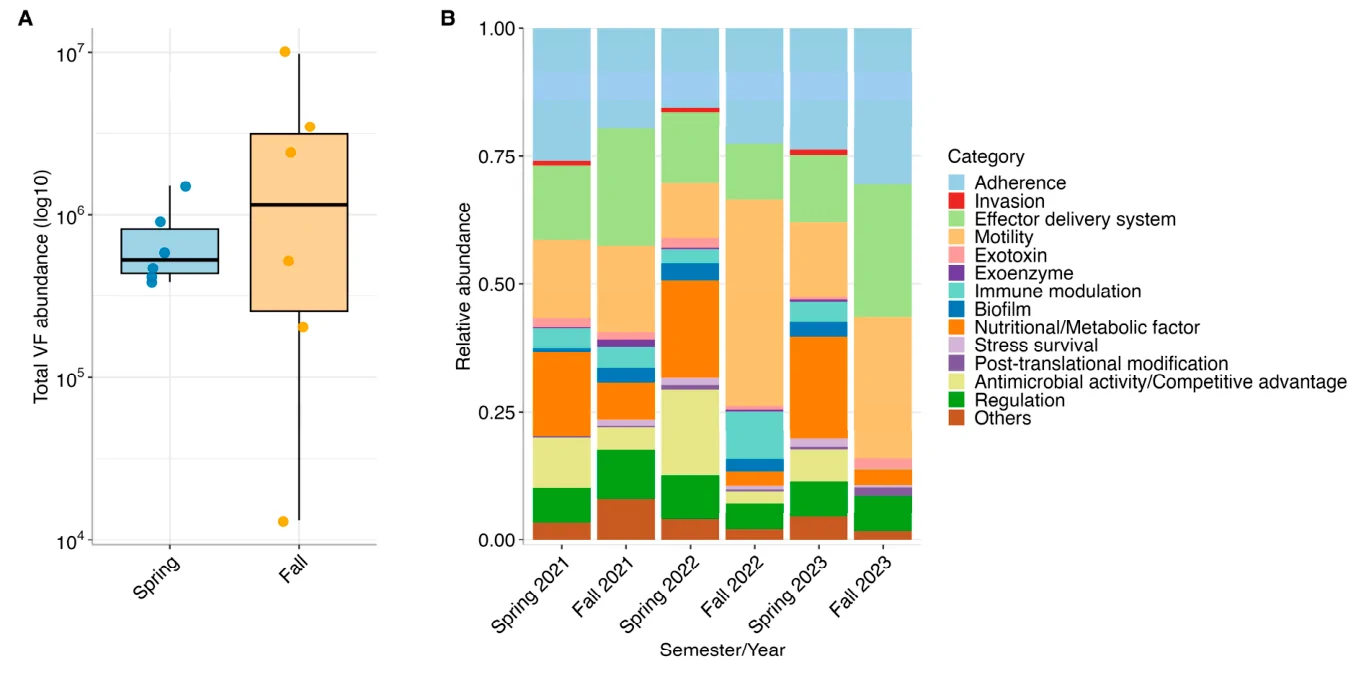
Virulence factor (VF) abundance and functional composition across semesters. (**A**) Total VF abundance (log_10_-transformed) by semester. Boxplots display the median and interquartile range; individual points represent each sampling date. Groups were compared using a Wilcoxon rank-sum test (W = 15, *p* = 0.699). (**B**) Mean relative abundance of 14 VF functional categories across semester/year groups (Spring and Fall, 2021–2023).

**Figure 7:**
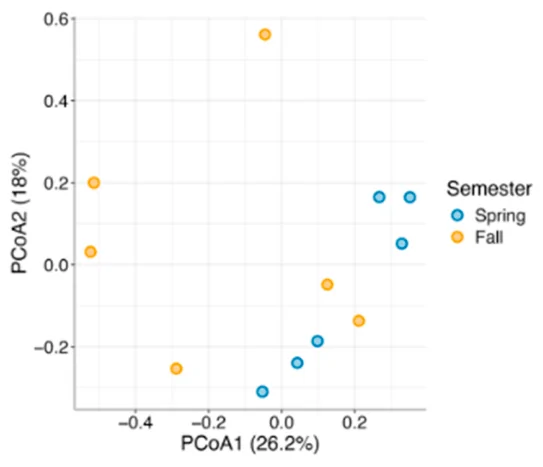
Virulence ordination. (PCoA) of gene-level VF profiles based on Bray–Curtis dissimilarities (PERMANOVA, R^2^ = 0.143, *p* = 0.069).

Total VF abundance was higher in Fall samples (median: 1.52 × 10^6^; IQR: 2.88 × 10^6^) compared to Spring samples (median: 5.34 × 10^5^; IQR: 3.93 × 10^5^), though this difference was not statistically significant (Wilcoxon rank-sum test, W = 15, *p* = 0.699; Figure 6A). Despite this difference in magnitude, VF functional composition remained broadly consistent across semesters (Figure 6B). On average, adherence (23.0 ± 5.5%), motility (20.9 ± 15.2%), effector delivery systems (16.9 ± 8.9%), and nutritional/metabolic factors (11.4 ± 11.2%) accounted for the largest proportions of virulence-associated traits across sampling periods. Minor categories were detected intermittently at low relative abundance: invasion (0.5 ± 0.9%), exotoxin (1.4 ± 1.1%), exoenzyme (0.5 ± 0.6%), immune modulation (4.0 ± 4.7%), and antimicrobial activity/competitive advantage (6.6 ± 7.6%). No individual category differed significantly between semesters after FDR correction (all adjusted *p* > 0.19). Gene-level profiles reflected these functional patterns, with a subset of VF-associated genes accounting for a disproportionate share of total VF abundance across semesters (Figure S5). Temporal variation in specific functional categories was observed; for example, Fall 2022 samples had higher contributions of motility-associated functions, whereas Spring 2022 samples had increased representation of antimicrobial activity/competitive advantage and stress-survival functions. Indicator species analysis identified three VF genes significantly associated with Spring samples: *yagW/ecpD* (adherence; stat = 0.709, *p* = 0.021), *kpsE* (immune modulation; stat = 0.680, *p* = 0.047), and *clbG* (antimicrobial activity/competitive advantage; stat = 0.547, *p* = 0.047); however, none remained significant after Benjamini–Hochberg FDR correction (all adjusted *p* > 0.05). No genes were significantly associated with Fall samples, consistent with the higher within-group variability observed in Fall samples (betadisper, F = 2.047, *p* = 0.182). PCoA suggested partial separation of samples by semester (Figure 7), although clustering was less distinct than for ARG profiles. PERMANOVA indicated that semester accounted for 14.3% of the variation in VF composition but was not statistically significant (R^2^ = 0.143, *p* = 0.069). Overall, VF profiles exhibited modest temporal structuring with substantial overlap between Spring and Fall samples.

### 2.5. Ecological Associations Between Resistome and Virulome Profiles

**Figure 8:**
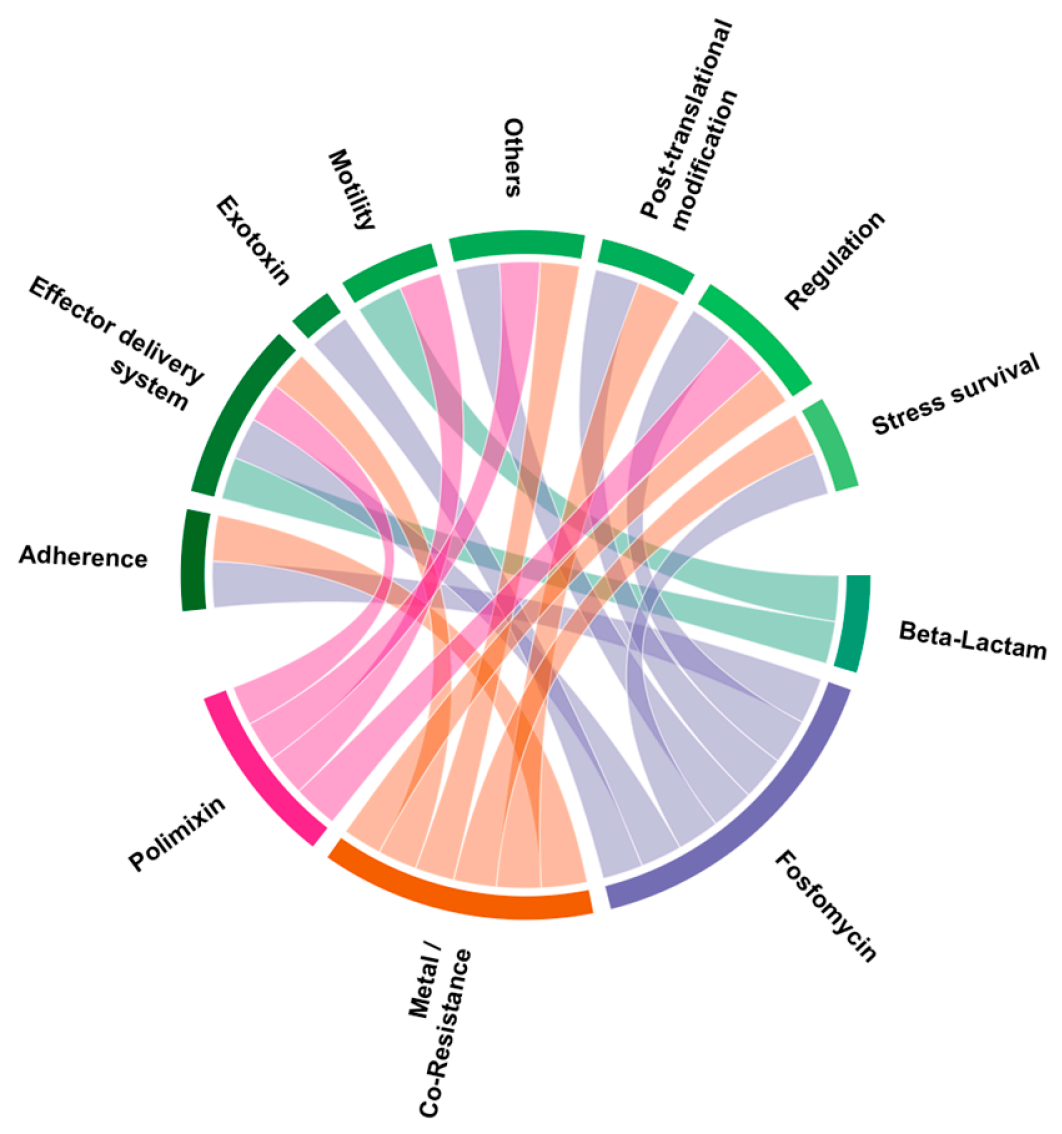
Chord diagram illustrating significant co-occurrence associations between antibiotic resistance gene (ARG) classes and virulence factor (VF) functional categories across wastewater samples. Ribbon colors correspond to ARG classes (blue = Beta-Lactam, purple = Fosfomycin, orange = Metal/Co-Resistance, pink = Polymyxin). VF category sectors are shown in green. Ribbon width is proportional to Spearman ρ. Only statistically significant associations (adjusted *p* ≤ 0.05, Benjamini–Hochberg correction) are shown. Fosfomycin and Metal/Co-Resistance showed the broadest connectivity, linking to the most VF functional categories.

Significant associations were observed between ARG classes and VF functional categories (Figure 8 and Figure S6). Positive correlations were detected among beta-lactam and polymyxin classes and VF categories related to regulation, effector delivery systems, and motility (Spearman ρ = 0.769–0.888, adjusted *p* ≤ 0.040). Metal/co-resistance and fosfomycin classes showed the broadest and strongest positive associations with virulence-related functions, including adherence, post-translational modification, regulation, stress survival, effector delivery systems, exotoxin, and the ‘Others’ VF category (ρ = 0.755–0.897, adjusted *p* ≤ 0.043, Figure S7; bootstrap 95% confidence intervals for all significant pairs are reported in Table S2). In contrast, no significant correlations were detected between biofilm formation, immune modulation, invasion, or nutritional/metabolic functions and any ARG class after correction for multiple comparisons. The overall correlation structure (Figure S8) indicated that most ARG–VF pairwise relationships did not reach statistical significance after BH correction; however, among those that did, correlations were predominantly strong (ρ = 0.755–0.897), particularly for fosfomycin and metal/co-resistance classes. Hierarchical clustering revealed grouping patterns among resistance classes and virulence functions with similar association profiles, consistent with shared ecological drivers influencing their co-variation across samples.

### 2.6. Co-Occurrence Patterns Among Microbial Taxa, ARG Classes, and Virulence Functions

**Figure 9:**
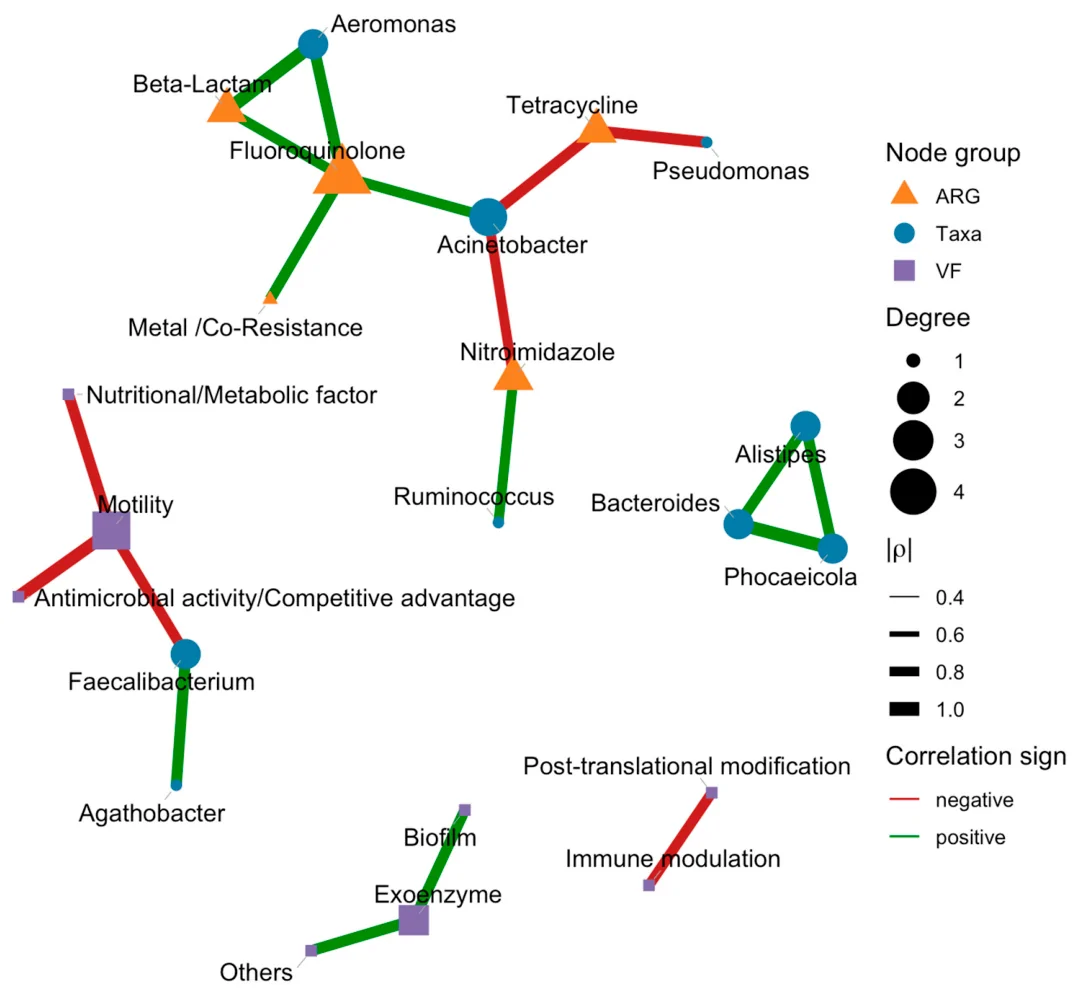
Co-occurrence network of microbial taxa, ARG classes, and virulence factor (VF) categories across wastewater samples. Edges represent Spearman rank correlations (|ρ| ≥ 0.40) that remained significant after Benjamini–Hochberg multiple-testing correction (adjusted *p* ≤ 0.05), computed on log1p-transformed relative-abundance profiles. All associations surviving correction are shown (no node exceeded the eight-edge-per-node display cap); analyses were restricted to the 15 most prevalent genera, 13 ARG classes, and 13 VF categories. Node shape denotes feature type (circles, taxa; triangles, ARG classes; squares, VF categories); node size is proportional to degree; edge color indicates correlation direction (green, positive; red, negative), with line width proportional to |ρ|. Of the 19 retained associations, seven were ARG–taxa, five VF–VF, four taxa–taxa, two ARG–ARG, and one taxa–VF; no direct ARG–VF associations survived correction. *Acinetobacter* and fluoroquinolone resistance exhibited the highest betweenness centrality.

Network analysis of the combined taxa–ARG–VF correlation matrix, retaining associations with |ρ| ≥ 0.4 that remained significant after Benjamini–Hochberg correction, produced a sparse, modular structure of 19 edges (Figure 9). No feature was connected to more than four others (maximum degree = 4), and the network resolved into several small, disconnected modules rather than a single connected structure. Resistance was organized primarily through bacterial taxa: ARG–taxa associations were the most frequent edge type (7 of 19) and formed the largest component, in which *Acinetobacter* and *fluoroquinolone* resistance had the highest betweenness centrality, linking beta-lactam, tetracycline, and nitroimidazole resistance to genera including *Aeromonas*, *Pseudomonas*, and *Ruminococcus*. A separate module comprised the gut-associated anaerobes *Bacteroides*, *Phocaeicola*, and *Alistipes* (4 taxa–taxa edges). Virulence functions co-varied predominantly with one another (5 VF–VF edges) rather than with resistance genes, forming small clusters around motility, exoenzyme production, and post-translational modification, with only a single taxon–VF edge retained. Notably, no direct ARG–VF associations survived correction. Within a single correlation family spanning all three feature types, resistance classes were therefore organized around bacterial taxa, and virulence functions formed their own co-varying groups, but the two domains did not couple directly at the community level.

### 2.7. Campus COVID-19 Testing and Quarantine Trends

**Figure 10:**
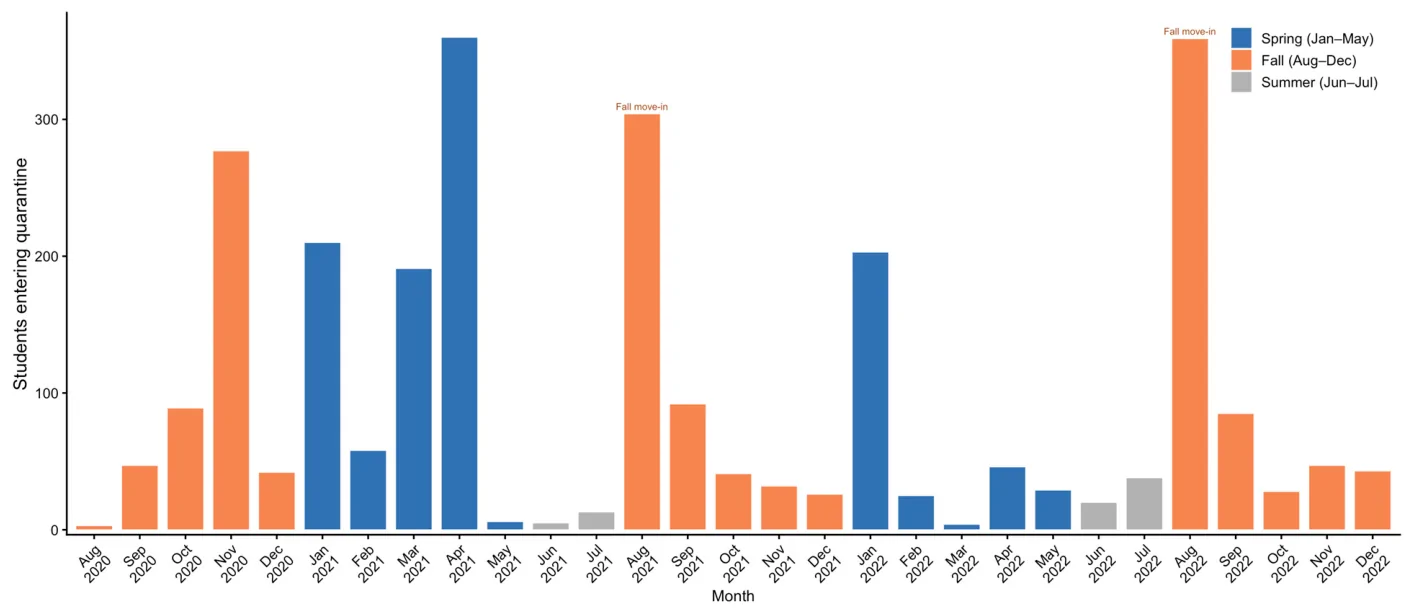
Monthly counts of students entering university-managed quarantine at North Carolina A&T State University (August 2020–December 2022), based on de-identified Student Health Center records.

Campus-level Student Health Center records provided temporal context for the sampling period. Between August 2020 and December 2022, 2771 COVID-19 testing and quarantine events were recorded, the majority among students (76%), followed by staff, athletes, and faculty. Quarantine activity was strongly seasonal, with pronounced peaks at the start of each fall semester, coinciding with student move-in (359 quarantine entries in August 2022 and 304 in August 2021; Figure 10), together with a spring peak in April 2021 (360 entries). The median quarantine duration was 10 days (IQR: 10–10 days), consistent with institutional isolation guidance during this period. These records ended in December 2022 and therefore did not extend to the 2023 samples.

## 3. Discussion

Wastewater from a building-scale quarantine residence reflects the interplay between human occupancy, antimicrobial exposure, and microbial community ecology. To our knowledge, this is among the first studies to resolve the joint ecological organization of taxa, ARGs, and VFs longitudinally within a single, defined human wastewater catchment at a COVID-19 quarantine residence hall. By integrating taxonomic composition with antimicrobial resistance and virulence profiles across semesters, this study reveals a layered functional structure within a semi-closed population. A persistent fecal core microbiome underpins the system, upon which temporally structured resistance patterns and comparatively stable virulence functions are superimposed. These findings illustrate how resistance and virulence traits are embedded within community ecology, yet differentially responsive to selective pressures operating at the building level.

### 3.1. Core Microbiome Stability Despite Temporal Variation

The consistent dominance of gut-associated anaerobic bacteria across all sampling periods reflects the residential character of the sewer catchment, where continuous fecal inputs from a defined student population constitute the primary microbial source. This pattern is consistent with previous wastewater metagenomic studies showing that human fecal microbiota are the primary drivers of microbial community structure in building-level sewer systems [4]. In contrast, wastewater systems receiving substantial stormwater inflow or industrial discharges often shift toward non-gut, environmental, or sewer-associated microbial taxa, reflecting input-driven changes in community composition [13,14]. The obligate and facultative anaerobes that dominate the human gut are continuously introduced into wastewater through fecal inputs and are well adapted to nutrient-rich wastewater environments, enabling their persistent detection despite fluctuations in temperature, hydraulic flow, and building occupancy [15,16]. The prevalence of these taxa therefore reinforces the concept that residence hall wastewater provides a reliable, stable population-level signal of the human-associated microbiome, supporting its utility for building-scale surveillance.

Despite this stability, Fall semester communities were more variable and showed a lower relative contribution of gut-associated genera than in Spring, a pattern confirmed by beta diversity analyses, which showed a significant difference in community structure between semesters. The greater dispersion of Fall samples in ordination space suggests that Fall communities are not only compositionally distinct from Spring but also more heterogeneous among themselves, likely reflecting the variability of early-semester dynamics, including fluctuating move-in occupancy, variable water usage, and heightened infection exposure at the start of the academic year [9,17]. The marked increase in *Acinetobacter* during Fall 2023 is particularly notable in this quarantine-dormitory setting. *Acinetobacter* species are well-recognized opportunistic pathogens with high environmental persistence and tolerance to disinfectants [18,19], and their elevated abundance in building-scale wastewater has previously been associated with increased clinical activity and antimicrobial selection pressure [20]. This transient bloom may therefore represent a population-level signal of altered infection dynamics or intensified disinfectant exposure within the dormitory during that period; a hypothesis that warrants direct investigation in future studies. The intermittent detection of *Klebsiella* and *Pseudomonas* across sampling periods is also noteworthy, as both genera are well-known carriers of clinically relevant ARGs, and their sporadic presence may reflect transient selective pressures within the catchment.

The significant year × semester interaction is the strongest structural signal in the community data and indicates that seasonal patterns in community composition were not consistent across years. This suggests that recurring seasonal dynamics, such as the transition between Spring and Fall occupancy, were modulated each year by non-repeating, year-specific perturbations, including COVID-19 infection waves, evolving quarantine protocols, and shifting antimicrobial use patterns throughout the study period [11,12]. The fact that year alone did not significantly explain community variation, whereas the interaction term explained the majority of the variation, underscores that neither season nor year operates independently; rather, their convergence shapes community structure in this system. Together, these findings support a model in which the residence hall wastewater microbiome is organized around a stable fecal core that persists across all sampling periods, upon which temporally and annually modulated variation in community structure is superimposed [15,16]. These microbial dynamics provide essential ecological context for interpreting patterns in the resistome and virulome.

### 3.2. Temporal Drivers of Resistome and Virulome Variation

The temporal structure of the resistome mirrored the patterns observed in microbial community composition, with seasonal shifts evident in the relative abundance of resistance classes despite overall stability in total ARG abundance [21]. This decoupling, in which total ARG abundance remained stable while the compositional structure shifted significantly between semesters, suggests that temporal resistome dynamics in this system are driven primarily by compositional turnover among resistance gene classes rather than by changes in the overall resistance burden. This pattern is consistent with longitudinal wastewater metagenomic evidence that resistome composition can shift over time, even as overall resistance gene abundance remains relatively stable, with established resistance genes persisting and attenuating only slowly [22]. This turnover suggests that the dominant resistance classes are sustained by persistent gut-associated bacteria across all sampling periods, whereas less stable resistance genes reflect the transient appearance of opportunistic taxa and episodic antimicrobial selection pressures [23,24]. The dominance of macrolide and tetracycline resistance across all sampling periods reflects continuous and non-episodic selective pressure from community-level antibiotic use, consistent with the ubiquity of these resistance classes in residential wastewater systems globally [4].

The elevated representation of beta-lactam, sulfonamide, and metal/co-resistance genes in Fall 2023 likely reflects the marked increase in *Acinetobacter* observed during that period, an organism well recognized for its capacity to harbor and disseminate clinically relevant beta-lactam resistance determinants. The concurrent elevation of metal- and co-resistance genes accompanied this shift, producing a coordinated enrichment of resistance determinants in Fall 2023. Co-selection between metal tolerance and antibiotic resistance is well documented in built-environment settings [9,17], making intensified disinfectant use a plausible contributor; however, disinfectant residual measurements and building-level antibiotic consumption data were not available to evaluate this directly. In contrast to the largely non-directional fluctuations in most classes, the significant directional decline in nitroimidazole resistance over the study period may reflect reduced clinical use of metronidazole or shifts in the anaerobic gut-associated community members that typically carry such resistance determinants, though this interpretation requires corroboration from clinical prescribing data.

In contrast to the resistome, the functional composition of VFs was broadly conserved across semesters, with adherence, effector delivery systems, motility, and nutritional or metabolic factors consistently representing the dominant virulence-associated categories. This relative stability suggests that virulence-associated traits are structurally embedded within the persistent fecal core microbiome rather than being driven by episodic community perturbations. The consistent representation of these functional categories across all sampling periods aligns with the ecological interpretation that the gut-associated anaerobic taxa dominating this system maintain a stable repertoire of colonization and host-interaction traits that persist despite seasonal shifts in community structure. The nominal Spring-specific enrichment (prior to FDR correction) of *yagW/ecpD*, which mediates pilus-based adherence and biofilm formation [25], *kpsE*, which facilitates capsular immune evasion [26], and *clbG*, which is required for colibactin genotoxin synthesis [27], suggests that commensal and potentially pathogenic *E. coli* strains carrying these traits were more abundantly represented during Spring, consistent with the higher overall contribution of gut-associated taxa during those periods and the tighter, more homogeneous community composition observed in Spring samples. The more modest temporal structuring of virulence profiles compared with resistance profiles further reinforces the distinction between virulence, which appears anchored to the stable community core, and resistance, which is more dynamically responsive to external selective pressures operating at the building scale.

### 3.3. Ecological Coupling Between Resistance and Virulence

The significant ecological coupling observed between specific ARG classes and virulence functional categories indicates that resistance and virulence determinants do not co-vary independently within this system but instead respond to shared ecological and genetic drivers. Positive associations between beta-lactam, polymyxin, metal/co-resistance, and fosfomycin resistance classes and functions related to motility, effector delivery systems, regulation, and adherence suggest coordinated variation in resistance and colonization-associated traits across samples. The overall magnitude of significant correlations was strong, particularly for fosfomycin and metal/co-resistance classes, which showed the broadest associations with virulence-related functions consistent with the influence of environmental stressors in shaping composite adaptive phenotypes in wastewater ecosystems. However, because most ARG–VF pairwise relationships did not reach statistical significance after multiple-comparison correction, stronger ecological coupling appears to be restricted to specific functional interactions rather than representing a genome-wide association across all resistance and virulence categories.

These coupled patterns may arise through several non-mutually exclusive mechanisms, which we outline as hypotheses. First, AMR and virulence genes frequently co-occur within mobile genetic elements, including plasmids, integrons, and transposons, which could facilitate their joint dissemination under selective pressure [28,29]. Second, co-selection mediated by antimicrobial exposure, disinfectants, or metal stress can enrich bacterial populations harboring both resistance and persistence-associated traits, as bacteria carrying plasmids with biocide- or metal-resistance genes are frequently also carriers of ARGs through co-resistance mechanisms [30]. Third, shifts in dominant taxa, particularly environmentally resilient and opportunistic pathogens such as *Pseudomonas*, *Acinetobacter*, and members of the *Enterobacteriaceae*, can simultaneously alter both resistome and virulome profiles through taxonomic turnover rather than direct genetic linkage [7,31]. Our correlational, short-read data cannot distinguish physical genetic linkage, such as co-localization of ARGs and VFs on the same mobile genetic element, from co-variation driven by shared host taxa; resolving this would require long-read sequencing or plasmid-resolved approaches beyond the scope of the present study.

These findings support a model in which building-scale wastewater systems act as ecological reservoirs where resistance and virulence traits are linked through overlapping selective forces and shared host populations. Temporal variation in antimicrobial exposure, population dynamics, and environmental conditions may reorganize the composition of resistance genes, while virulence-associated functions provide a comparatively stable ecological framework within which these changes occur. The resulting system reflects neither complete independence nor strict coupling, but rather a form of modular ecological integration between resistance and virulence traits in a human-impacted environment. Consistent with this, the direct ARG–VF associations identified in the pairwise analysis did not persist in the joint co-occurrence network, where correction was applied across all taxa, ARG, and VF comparisons; the two analyses are therefore complementary, indicating resistance–virulence coupling at the level of specific class–function pairs rather than as community-wide connectivity.

### 3.4. Network Structure Reflects Taxa-Anchored Modular Organization

The ecological organization of the wastewater microbiome extends beyond pairwise associations to a higher-order network structure in which resistance and virulence traits are embedded within coordinated functional assemblages shaped by shared environmental pressures and host-associated inputs. After correction for multiple comparisons, the structure was sparse and modular, resolving into several small, disconnected components. This is consistent with ecological filtering and functional specialization in microbial co-occurrence networks across wastewater and other engineered systems [32,33,34].

Within this structure, resistance was organized around bacterial taxa; *Acinetobacter* and fluoroquinolone resistance showed the highest betweenness centrality and anchored the largest component, linking beta-lactam, tetracycline, and nitroimidazole resistance to genera including *Aeromonas*, *Pseudomonas*, and *Ruminococcus*. The prominence of *Acinetobacter* is consistent with its recognized role as an environmentally resilient opportunistic pathogen that carries broad ARG profiles [35,36]. A separate module of gut-associated anaerobes, *Bacteroides*, *Phocaeicola*, and *Alistipes*, reflects their consistent and abundant presence within the stable resident fecal community [15,17]. Virulence functions, in turn, co-varied predominantly with one another rather than with resistance genes, forming small clusters; more weakly connected functions such as adherence, stress survival, and exotoxin production likely represent specialized or transient community members contributing to narrower functional niches [34].

Notably, no direct ARG–VF associations survived correction within the joint network. This indicates that, at the whole-community level, resistance and virulence determinants are not broadly co-connected but are instead organized into semi-independent modules that respond to similar selective pressures. Such modular organization suggests that resistance and virulence traits are structured into functional guilds associated with specific ecological strategies, such as host interactions, environmental persistence, or metabolic adaptation, rather than into a single interconnected assemblage. The predominance of positive associations and the relatively few strong negative correlations further suggest that cooperative or co-selected processes, rather than competitive exclusion, play a dominant role in shaping these relationships, consistent with environments characterized by continuous microbial input and overlapping ecological niches.

Together, these observations indicate that the wastewater microbiome is organized into modular structures in which resistance and virulence traits are linked to bacterial taxa and shared selection pressures. This organization has implications for gene dissemination, as taxa that carry resistance determinants and persist across conditions, most prominently *Acinetobacter*, may act as reservoirs that facilitate the persistence of clinically relevant resistance within human-impacted environments.

### 3.5. Implications for Building-Scale Wastewater Surveillance

Building-scale wastewater monitoring has emerged as a powerful approach for capturing microbial and genetic signals associated with localized human populations [4]. The results of this study demonstrate that wastewater metagenomics can extend beyond pathogen detection to characterize the ecological organization of antimicrobial resistance and virulence traits within confined built environments. The detection of diverse resistance classes and virulence-associated functions across sampling periods highlights the role of wastewater systems as reservoirs of microbial functional genes that reflect both antimicrobial exposure and host-associated inputs. At the scale of individual buildings, wastewater captures signals from well-defined populations, enabling the detection of localized shifts in resistome and virulome composition that may be obscured in larger municipal systems where signals are diluted [9]. This higher spatial resolution has important implications for surveillance. Building-level monitoring can support early detection of emerging resistance patterns and changes in virulence-associated functions, particularly in settings such as university residences, healthcare facilities, or long-term care environments where transmission dynamics are spatially structured [37,38]. The temporal variability observed in resistance composition, coupled with the relative stability of core virulence functions, suggests that wastewater surveillance may be sensitive to shifts in antimicrobial use and environmental pressures, yet still capture consistent indicators of pathogenic potential. Importantly, integrating taxonomic, resistance, and virulence data provides a more comprehensive framework for interpreting wastewater signals. Network-based analyses further indicate that resistance genes and virulence traits are nonrandomly associated, organized within structured ecological relationships, allowing the identification of potential microbial hosts, functional modules, and co-occurring gene assemblages. Such insights can improve the interpretation of surveillance data by linking observed gene patterns to underlying ecological processes rather than treating them as independent markers.

From an applied perspective, these findings support the potential of high-resolution wastewater metagenomics as a complementary surveillance tool for monitoring AMR in semi-contained populations. Building-scale surveillance can identify emerging resistance signals, characterize temporal shifts in resistance profiles, and help prioritize potential hotspots for targeted follow-up [39,40]. Such surveillance could strengthen public health situational awareness by providing early indications of changes in the local resistome; however, translating these signals into antimicrobial stewardship or infection control actions would require integration with clinical, epidemiological, and prescribing data, which were not available in the present study. The β-lactam resistome illustrates this potential by linking the ecological organization described above to a clinically consequential signal. Serine β-lactamases accounted for the overwhelming majority of β-lactam resistance gene abundance, and within Class A enzymes, carbapenemase genes, driven primarily by *bla*KPC, were severalfold more abundant than individual extended-spectrum β-lactamase genes. The detection of a *bla*KPC-dominated carbapenemase signal within a single building catchment is notable as a surveillance observation, given the recognized clinical importance of carbapenem-resistant *Enterobacterales* and KPC-type enzymes in treatment-limiting resistance [41]. This signal is sequence-based and was not confirmed by culture or PCR; it therefore indicates the presence of carbapenemase gene sequences in wastewater rather than confirmed viable resistant organisms and supports targeted confirmatory follow-up. Beyond this static picture, the β-lactam resistome was temporally dynamic: the *Amp*C-type cephalosporinases *bla*CMY and *bla*MOX were significantly enriched in Fall semesters, coincident with *Acinetobacter*-associated community shifts, and *bla*CMY showed a significant, progressive increase across the study period, independent of seasonal variation. These observations indicate that building-scale metagenomic surveillance can resolve both the presence and the temporal trajectory of resistance determinants of clinical interest at the level of individual gene subtypes, a resolution that could support future stewardship and infection control applications when integrated with clinical data. Building on this temporal resolution, longitudinal monitoring at this scale may enable tracking of intervention outcomes and early warning of emerging resistance threats before they become widespread within communities [22,42]. Together, this study highlights the potential of building-scale wastewater surveillance as a sensitive and scalable approach to monitoring AMR and virulence dynamics in human-impacted environments. Integrating ecological interpretation with routine surveillance frameworks may enhance our ability to detect, understand, and respond to microbial risks in real time.

### 3.6. Limitations and Future Directions

While this study advances understanding of resistance–virulence co-organization in building-scale wastewater, several considerations define priorities for future research. The modest sample size across six semester-year periods limits statistical power and resolution of temporal trends and warrants cautious interpretation of multivariate and network analyses. With twelve samples as the unit of replication, multivariate models fitted across multiple predictors are also susceptible to overfitting, and the variance explained by the year × semester interaction is likely to be optimistic and imprecisely estimated. Because the Benjamini–Hochberg procedure controls the FDR across multiple comparisons, associations that remain significant after correction provide more stringent evidence, while results near the significance threshold should be interpreted with appropriate caution.

The reliance on single daytime grab samples, rather than 24 h flow-weighted composites, means that diurnal residential activity and hydraulic variability may influence individual measurements. In addition, the a priori preference for sampling dates with SARS-CoV-2 positivity may have biased the sampled periods toward higher occupancy or infection pressure, potentially influencing temporal comparisons of microbial community, ARG, and VF profiles. Campus occupancy and infection pressure records ended in December 2022 and therefore do not extend to the 2023 samples; consequently, the Fall 2023 community and resistome changes are interpreted without contemporaneous occupancy context and are not attributed specifically to occupancy dynamics. Wastewater flow, continuous occupancy counts, and a fecal-strength normalization marker (e.g., crAssphage or PMMoV) were also unavailable, so some observed shifts may partly reflect dilution or population-size changes rather than biological turnover. Fecal-strength normalization should therefore be incorporated into future building-scale studies.

Although shotgun metagenomics enables simultaneous profiling of microbial taxa, ARGs, and VFs, the gene-level profiling used here does not, by itself, establish which host organisms carry these genes; host assignment would require complementary approaches such as metagenome-assembled genome reconstruction or long-read sequencing. Sequencing-based methods also detect genetic material regardless of viability; therefore, detected ARGs and VFs may not represent actively expressed genes or viable pathogens.

Finally, sampling within a single residential building provides the high spatial resolution that is a central strength of the building-scale approach but limits the broader generalizability of the observed patterns. Future studies incorporating longitudinal multi-site sampling across diverse building types, together with host-resolved genomics, transcriptomics, and antimicrobial prescribing metadata, would help establish the generalizability of these findings, strengthen interpretation of resistance patterns, and evaluate the potential translation of building-scale wastewater surveillance into public health applications.

## 4. Materials and Methods

### 4.1. Sampling Site and Collection

**Figure 11:**
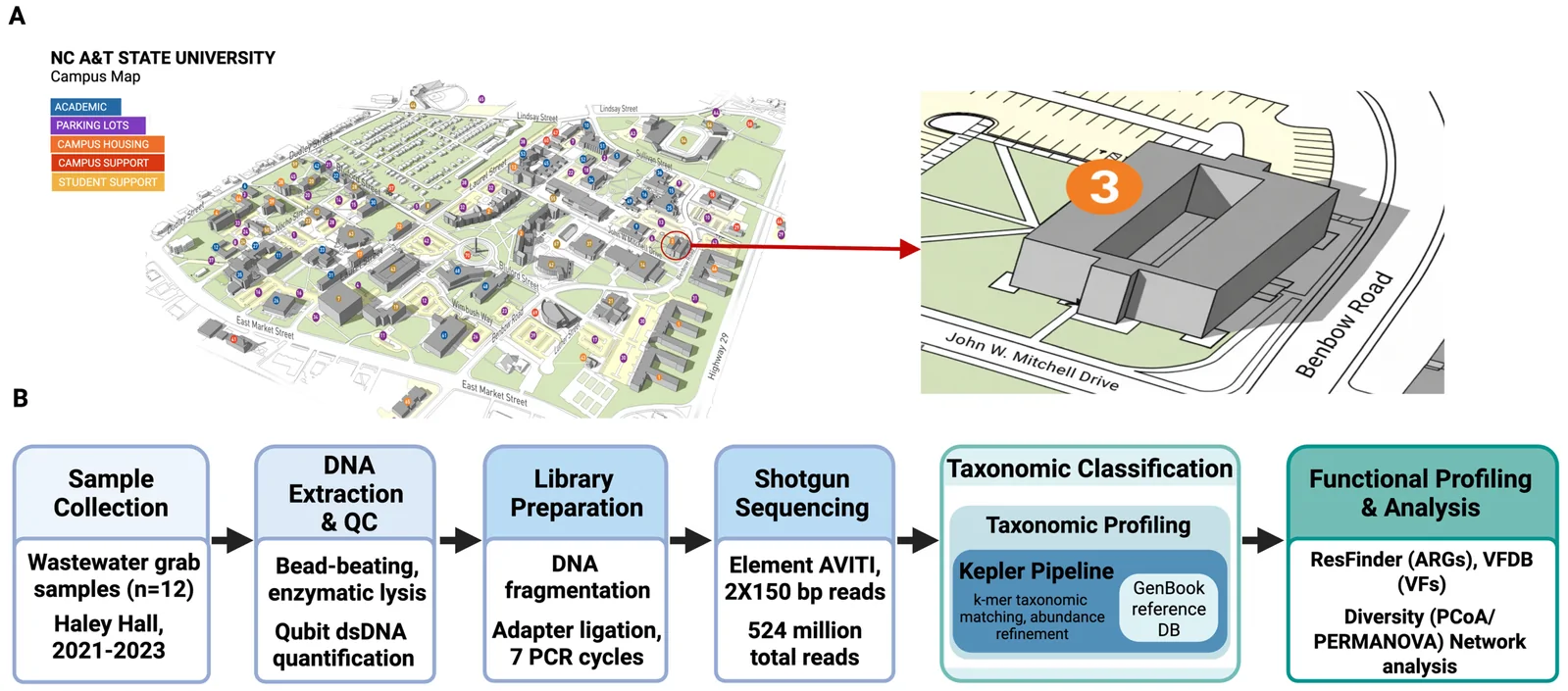
Study design and metagenomic workflow. (**A**) Location of Haley Hall within North Carolina A&T State University campus (inset) with a detailed view of the wastewater sampling location used for longitudinal metagenomic analysis. (**B**) Overview of the experimental and bioinformatic workflow. Created in BioRender. Morikwe, U. (2026) https://BioRender.com/d73ley0.

A longitudinal wastewater metagenomics study was conducted at North Carolina A&T State University (Greensboro, NC, USA) to assess temporal patterns in microbial community composition, ARGs, and VFs. As part of a broader campus wastewater surveillance program, samples were collected biweekly from the manholes of 11 designated residence halls (Figure 11) between January 2021 and December 2023 [43]. Sampling was performed over a three-year period (2021–2023) during the spring and fall academic semesters to capture seasonal and interannual variability. For the present study, analyses focused on samples collected from a COVID-19 quarantine residence hall (Haley Hall), which housed students isolating after positive diagnoses and therefore represented a defined human-associated wastewater catchment. Grab samples were collected during routine daytime building operations to ensure consistency in occupancy-associated wastewater inputs. A subset of twelve samples (two per semester) was selected a priori for shotgun metagenomic sequencing to provide balanced temporal coverage across semesters (Table S3). Preference was given to samples with confirmed SARS-CoV-2 RNA detection, while maintaining representation across the study period and ensuring that DNA quantity/quality requirements were met; the twelve samples therefore constitute a purposively selected, rather than random, subset of the biweekly series. Each sample represented an independent wastewater grab sample collected at a unique time point, distributed across six semester–year periods spanning 2021–2023 (Table S3), rather than drawn as consecutive biweekly samples from a single interval. Of the twelve samples, ten were confirmed positive for SARS-CoV-2 RNA by RT-qPCR [43] as part of the campus wastewater surveillance program. Samples were collected in sterile containers and transported on ice; procedures were conducted under institutional biosafety approval (North Carolina A&T State University IBC protocol #20-11).

### 4.2. Campus COVID-19 Testing and Quarantine Records

To provide population-level context for the wastewater sampling period, COVID-19 testing and quarantine records were obtained from the North Carolina A&T State University Student Health Center under Institutional Review Board (IRB) approval protocol HS26-0058. These campus-level records included the date of COVID-19 testing, entry and exit dates for university-managed quarantine, and basic demographic descriptors (affiliation and gender) for individuals processed through the health center between August 2020 and December 2022. Because the residence hall examined in this study (Haley Hall) served as a designated quarantine facility, the number of individuals entering quarantine each month was used as a proxy for building occupancy and infection pressure during the sampling period. Quarantine duration was calculated as the interval between recorded entry and exit dates. These records were used solely to describe temporal patterns of testing and quarantine activity; they were campus-wide and were not linked to antimicrobial prescribing, clinical diagnoses, or individual wastewater samples. Monthly and semester-level counts were summarized in R (v4.3.0) and presented descriptively.

### 4.3. DNA Extraction and Quality Assessment

Total genomic DNA extraction and downstream sequencing preparation were performed by a commercial sequencing provider (Cmbio, Germantown, MD, USA). Wastewater samples (approximately 50 mL per sample) were submitted for shotgun metagenomic sequencing, and DNA was isolated using the provider’s optimized (proprietary) extraction workflow, which incorporates mechanical bead-beating and enzymatic lysis techniques to ensure efficient recovery of microbial DNA from environmental samples. Following extraction, DNA concentration was quantified using a Qubit Flex fluorometer with the Qubit™ dsDNA High Sensitivity Assay Kit (Thermo Fisher Scientific, Waltham, MA, USA) prior to library preparation.

### 4.4. Metagenomic Sequencing and Analysis

Shotgun metagenomic sequencing was performed to profile microbial community composition and functional gene content, including ARGs and VFs.

#### 4.4.1. Metagenomic Library Preparation and Sequencing

Metagenomic libraries were prepared using the Watchmaker DNA Library Preparation Kit (7K0019-1K) (Watchmaker Genomics, Boulder, CO, USA). Genomic DNA was enzymatically fragmented using Watchmaker Frag/AT reagents, followed by end-repair and adapter ligation with IDT xGen™ Unique Dual Index primers and IDT Stubby adapters (Integrated DNA Technologies, Coralville, IA, USA). Libraries were amplified for seven PCR cycles and purified with CleanNGS magnetic beads (CleanNA, Waddinxveen, The Netherlands). Final library concentrations were quantified with the Qubit™ dsDNA High Sensitivity Assay. Libraries were circularized using the Element Adept compatibility workflow and sequenced on the Element AVITI platform (Element Biosciences, San Diego, CA, USA) using the AVITI 2× 150 bp Cloudbreak sequencing kit. Sequencing generated 17.3–88.9 million read pairs per sample (mean 43.6 million), totaling approximately 524 million read pairs across the 12 wastewater samples. Samples were sequenced using the same platform and under identical run conditions.

#### 4.4.2. Bioinformatic Processing and Taxonomic Classification

Taxonomic profiling was performed using the Kepler multi-kingdom metagenomic classification pipeline (CosmosID/Cmbio) [44,45], which combines k-mer-based identification with probabilistic alignment-based abundance estimation. In the database construction, high-quality microbial genomes were curated to generate the GenBook reference database [44], which includes approximately 30,000 species across Bacteria, Archaea, Fungi, Protists, and Viruses. Genomes were filtered based on assembly quality, completeness, and low contamination levels, and low-complexity regions, plasmids, prophages, and host-derived sequences were removed to maximize taxonomic signal, using the default quality-control and filtering thresholds implemented in the Kepler pipeline. Sequencing reads were split into k-mers and compared against the reference GenBook database; exact k-mer matches were used to rapidly identify candidate taxa and eliminate non-representative genomes. Abundance estimates were subsequently refined using a probabilistic Smith–Waterman alignment approach that compares sequencing reads to candidate reference taxa and resolves ambiguous assignments via iterative maximum-likelihood estimation, classifying taxa to subspecies resolution at an average nucleotide identity divergence below 0.003. The normalized abundance matrix generated by the Kepler pipeline was used for all downstream taxonomic analyses, including alpha diversity, beta diversity, ordination, clustering, and permutational multivariate statistical testing.

#### 4.4.3. Antimicrobial Resistance Gene and Virulence Factor Identification

ARGs and VFs were identified using the biomarker-based Kepler AMR/VF profiling framework implemented within the Cosmos-Hub platform, leveraging curated nucleotide sequence databases, including ResFinder for ARGs and the Virulence Factor Database (VFDB) for virulence genes [46,47]. Reference gene sequences were parsed into variable-length n-mers, which were then classified as shared or unique biomarkers. Shared n-mer biomarkers represent homologous or overlapping nucleotide sequences across multiple gene families and form the higher-level nodes of a hierarchical tree-like data structure, whereas unique n-mer biomarkers correspond to gene-specific sequences and define the terminal nodes. Metagenomic sequencing reads from each sample were independently partitioned into k-mer sets and queried against this structured biomarker database to identify exact matches between sample-derived k-mers and reference biomarkers. This exact matching strategy enhances specificity in distinguishing closely related gene families and reduces spurious assignments. Gene abundance was estimated using fine-grained composite k-mer statistics in combination with sequencing coverage depth. These metrics were integrated into weighted biomarker scores (abundance scores), which were normalized for sequencing depth and biomarker coverage to estimate ARG and VF abundances within each sample.

#### 4.4.4. Open-Source Validation

To provide independent, open-source validation of the closed-source Kepler/GenBook pipeline, representative samples were re-analyzed on the public Galaxy platform (use.galaxy.org v26.1.2) [48] using two tools recommended during peer review. Taxonomic composition was validated with Kraken2 (v2.1.7) on four representative samples spanning both seasons and two years (Fall 2021, Spring 2023, and both Fall 2023 samples). Paired-end reads were classified against the Kraken2 Standard prebuilt database (build: 2020-11-26T021706Z), using the default Galaxy Kraken2 classification parameters (confidence threshold = 0, minimum base quality = 0, and minimum hit groups = 2). Kraken2 recovered *Acinetobacter* as a dominant genus in both Fall 2023 samples (37.0% and 17.8%) but at substantially lower relative abundance in Spring 2023 (2.0%) and Fall 2021 (0.4%), supporting the Fall 2023 *Acinetobacter* expansion and its year-specific nature. Gut-associated taxa, including *Bacteroides*, *Faecalibacterium*, and *Phocaeicola*, were likewise prominent in Spring 2023 and Fall 2021 (Table S4). For ARG and VF validation, both Fall 2023 samples were assembled with MEGAHIT (v1.2.9) using paired-end reads and k-mer sizes of 21, 29, 39, 59, 79, 99, 119, and 141, with a minimum contig length of 200 bp. The resulting contigs were screened with ABRicate (v1.4.0) against the ResFinder and VFDB databases, the same reference databases used for ARG and VF identification in the primary analysis, using minimum nucleotide identity and coverage thresholds of 80%. ABRicate recovered the major β-lactam and AmpC determinants (*bla*CMY, *bla*MOX, *bla*FOX, *bla*ACT, *bla*PER, multiple *bla*OXA), together with fosfomycin, colistin (*mcr*), and the tetracycline, sulfonamide, aminoglycoside, macrolide, and fluoroquinolone resistance determinants, and VFs representing the dominant functional categories reported in the primary analysis, including motility, effector delivery systems, adherence, and nutritional/metabolic factors (Table S5). Because Kraken2 and Kepler use different reference databases and ABRicate operates on assembled contigs, comparisons assessed concordance in dominant taxa, presence of ARGs, and VF category representation rather than exact abundances. Tool versions, database selections, and analysis parameters used for open-source validation are reported in Tables S4 and S5.

### 4.5. Data Analysis and Visualization

Statistical analyses and visualization were conducted in R (v4.5.2) [49]. Taxonomic, ARG, and VF abundance matrices generated by the Kepler pipeline were used for diversity and multivariate analyses. Relative abundance profiles were calculated for compositional visualizations, including stacked bar plots. Beta-lactam ARG subtypes were classified by enzyme class (Class A, B, C, and D) and gene variant based on ResFinder database [46] as implemented within the Kepler AMR/VF profiling framework [44]; enzyme classes were assigned according to the Bush–Jacoby classification scheme [50]. Subtype-level abundance profiles were analyzed alongside class-level data to assess compositional diversity within the beta-lactam resistome. Alpha diversity (Shannon and richness indices) and beta diversity (Bray–Curtis dissimilarity) were calculated (vegan v2.7-5) [51] to assess microbial community structure. Differences in alpha diversity between semesters were evaluated using Wilcoxon rank-sum tests. Patterns of between-sample community structure were assessed using principal coordinates analysis (PCoA) based on Bray–Curtis dissimilarity, and differences in community composition between semesters and years were tested using permutational multivariate analysis of variance (PERMANOVA; 999 permutations). Differences in multivariate dispersion between semesters were evaluated using PERMDISP [51]. Indicator species analysis was performed using the multipatt function in the indicspecies package (v1.8.0) [52] to identify VF genes associated with Spring or Fall samples, with significance assessed by 999 permutations and Benjamini–Hochberg correction. For analyses involving multiple comparisons, *p*-values were adjusted using the Benjamini–Hochberg procedure to control the FDR. Statistical significance for these analyses was assessed using the adjusted *p*-values, with values < 0.05 considered statistically significant. Raw *p*-values were retained for individual tests not subject to multiple-comparison correction.

Co-occurrence relationships between ARG classes and virulence function categories were evaluated using Spearman rank correlation based on log1p-transformed normalized abundance profiles across wastewater samples. Dominant microbial genera were defined as those with the highest mean normalized abundance across all samples, whereas all detected ARG classes and VF categories were included in the analysis. Correlations were computed with the Hmisc package (v5.2-5) [53]; only associations with |ρ| ≥ 0.40 and adjusted *p* ≤ 0.05 were retained for network construction, and community (modular) structure was identified using the Louvain algorithm. Confidence intervals for significant ARG–VF correlations were estimated by bootstrap resampling with replacement (5000 replicates), using the percentile method (Table S2). Connector nodes were identified by degree and normalized betweenness centrality.

Significant ARG–VF associations were visualized in two complementary ways. Clustered heatmaps were generated using the pheatmap package in R (v1.0.13) [54], with hierarchical clustering applied to both ARG classes and VF categories based on the full Spearman correlation matrix; only BH-corrected significant associations (adjusted *p* ≤ 0.05) are displayed in the correlation heatmap. Significant associations were additionally visualized as a chord diagram using the circlize package in R (v0.4.18) [55], with ribbon width proportional to Spearman ρ and ribbons colored by ARG class to highlight the resistance classes with the broadest virulence associations. Scatter plots of the top six significant ARG–VF pairs were generated using ggplot2 to illustrate the strength and direction of individual associations (v4.0.1) [56].

Integrated microbiome–ARG–VF co-occurrence networks were constructed in R using igraph (v2.2.1) [57] and visualized as undirected networks with ggraph (v2.2.2) [58] and ggplot2 (v4.0.1) [56], with nodes representing genera, ARG classes, or virulence categories. Node size reflected degree, and edges were weighted by the absolute value of the correlation coefficient and colored by sign, allowing identification of network modules and connector nodes.

## 5. Conclusions

This study demonstrates that building-scale wastewater metagenomic assessment provides a high-resolution window into the ecological organization of microbial communities, AMR, and virulence traits within a confined human-associated environment. The wastewater microbiome of this university residence hall was anchored by a stable fecal core microbiome of gut-associated anaerobic bacteria across all sampling periods, upon which temporally and annually modulated variation in community structure, resistome composition, and virulence profiles was superimposed. Seasonal and year-specific perturbations, particularly the marked community shift during Fall 2023, shaped both the taxonomic and functional gene landscape. The coupling between specific resistance classes and virulence functions, together with the taxa-anchored modular structure of the co-occurrence network, indicates that these traits are not independently distributed but are organized around bacterial taxa and shared selective pressures. This organization has direct implications for how wastewater-derived signals should be interpreted, not as isolated genetic markers but as ecologically embedded indicators of microbial community state and functional potential. Together, these findings establish a framework for integrating ecological interpretation with building-scale wastewater surveillance, with potential applications for antimicrobial stewardship, infection control, and early warning of the emergence of resistance in semi-contained populations when integrated with clinical and prescribing data. As wastewater-based epidemiology continues to expand beyond pathogen detection, this work contributes to a growing body of evidence supporting its utility as a sensitive, non-invasive, and population-relevant tool for monitoring the microbial dimensions of public health.

## Data Availability

All sequencing data generated in this study are available through NCBI BioProject accession PRJNA1455368.

## Supplementary Materials

The following supporting information can be downloaded at https://www.mdpi.com/article/10.3390/antibiotics15090878/s1. Figure S1: Top 20 dominant bacterial species across semesters (2021–2023); Table S1: Wilcoxon rank-sum test results comparing relative abundance of antimicrobial resistance gene (ARG) classes between Spring and Fall semesters; Figure S2: Nitroimidazole decreasing trend; Figure S3: Relative contribution of β-lactam subgenes within enzyme classes (A, C, and D) across Spring and Fall semesters (2021–2023); Figure S4: Relative contribution of seven aminoglycoside subclass compositions by semester/year; Figure S5: Relative abundance of the top 20 virulence factor (VF) genes across semester/year groups (2021–2023); Figure S6: Significant correlations between ARG classes and VF functional categories; Figure S7: Scatter plots of the six strongest significant ARG classes and VF functional category correlations; Figure S8: Full correlation matrix between ARG classes and VF categories; Table S2: Spearman correlation coefficients (ρ) with bootstrap 95% confidence intervals for the ARG class–VF category pairs significant after Benjamini–Hochberg correction; Table S3: Collection dates of the twelve wastewater samples selected for shotgun metagenomic sequencing by academic semester and year; Table S4: Independent open-source taxonomic validation of the Kepler/GenBook classification; Table S5: Independent open-source ARG and VF validation for both Fall 2023 samples.

## Abbreviations

The following abbreviations are used in this manuscript:

Abbreviation	Definition
AMR	antimicrobial resistance
AmpC	AmpC-type β-lactamase (Ambler class C cephalosporinase)
ARG	antimicrobial resistance gene
AVITI	Element AVITI (sequencing platform)
BH	Benjamini–Hochberg (correction)
bp	base pair
CRE	carbapenem-resistant *Enterobacterales*
CV	coefficient of variation
COVID-19	coronavirus disease 2019
DNA	deoxyribonucleic acid
ESBL	extended-spectrum β-lactamase
FDR	false discovery rate
IBC	Institutional Biosafety Committee
IQR	interquartile range
IRB	Institutional Review Board
KPC	*Klebsiella pneumoniae* carbapenemase
MGE	mobile genetic element
NCBI	National Center for Biotechnology Information
PCoA	principal coordinates analysis
PCR	polymerase chain reaction
PERMANOVA	permutational multivariate analysis of variance
PERMDISP	permutational analysis of multivariate dispersions
qPCR	quantitative PCR
RNA	ribonucleic acid
RT-qPCR	reverse transcription quantitative PCR
SARS-CoV-2	severe acute respiratory syndrome coronavirus 2
SD	standard deviation
VF	virulence factor
VFDB	Virulence Factor Database
