# Supplementary material for: Building-Scale Wastewater Metagenomics Reveals Temporal Patterns in Resistance and Virulence Genes
Source: Antibiotics (Basel). 2026 Sep 8;15(9):878. doi: 10.3390/antibiotics15090878 (PMC13603407; doi:10.3390/antibiotics15090878)
Supplement: Supplementary file 1 [file antibiotics-15-00878-s001.zip › antibiotics-4501754-supplementary.pdf]

Supplementary Materials

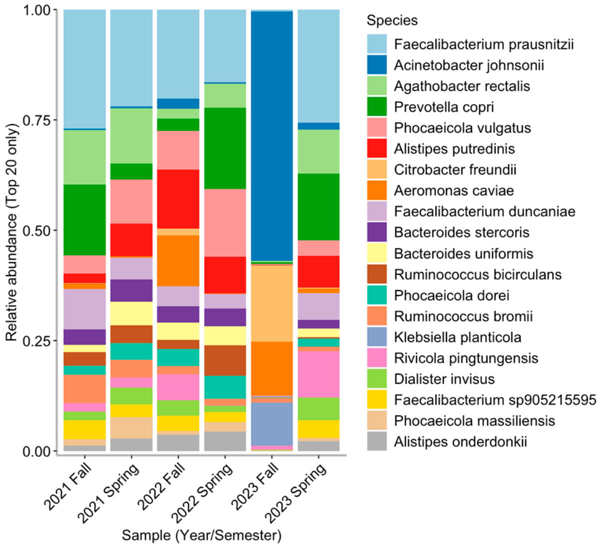

Figure S1. Top 20 dominant bacterial species across semesters (2021–2023)

Table S1. Wilcoxon rank-sum test results comparing relative abundance of antimicrobial resistance gene (ARG) classes between Spring and Fall semesters. Median and mean relative abundances (%) are shown for each group (n = 6 per semester). Raw p-values and Benjamini–Hochberg FDR-adjusted p-values are reported. No class reached significance after FDR correction (all q > 0.05). Tests were performed with normal approximation (exact = FALSE) to accommodate tied values.

**ARG Class**	**n (Spring)**	**n (Fall)**	**Median Spring (%)**	**Median Fall (%)**	**Mean Spring (%)**	**Mean Fall (%)**	**Raw p-value**	**FDR-adjusted p-value**
Beta-Lactam	6	6	5.283	14.909	5.272	17.074	0.0131	0.1175
Tetracycline	6	6	42.239	15.931	42.293	19.528	0.0202	0.1175
Metal /Co-Resistance	6	6	1.314	4.414	1.659	8.167	0.0306	0.1175
Fluoroquinolone	6	6	0.103	0.555	0.187	0.797	0.045	0.1175
Polymyxin	6	6	0.248	0.641	0.303	0.712	0.0453	0.1175
Fosfomycin	6	6	0.07	0.249	0.068	0.273	0.0542	0.1175
Aminoglycoside	6	6	3.837	8.919	5.122	11.041	0.1735	0.2856
Vancomycin	6	6	0	0	0.02	0	0.1757	0.2856
Sulfonamide	6	6	1.248	2.41	1.866	7.504	0.2298	0.3319
Macrolide	6	6	42.569	31.946	39.971	32.953	0.298	0.3521
Trimethoprim	6	6	1.483	0.663	2.238	0.992	0.298	0.3521
Nitroimidazole	6	6	0.123	0	0.167	0.051	0.3261	0.3532

Phenicol	6	6	0.738	0.582	0.835	0.91	0.4712	0.4712

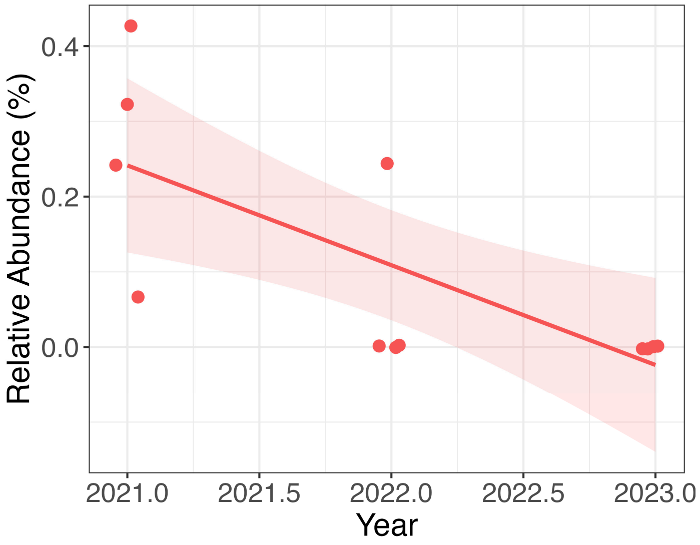

Figure S2. Nitroimidazole decreasing trend. Nitroimidazole relative abundance decreased significantly over time (Spearman's ϱ = −0.79, Benjamini–Hochberg adjusted p = 0.028). The solid line represents the linear regression fit, with the 95% confidence interval shown.

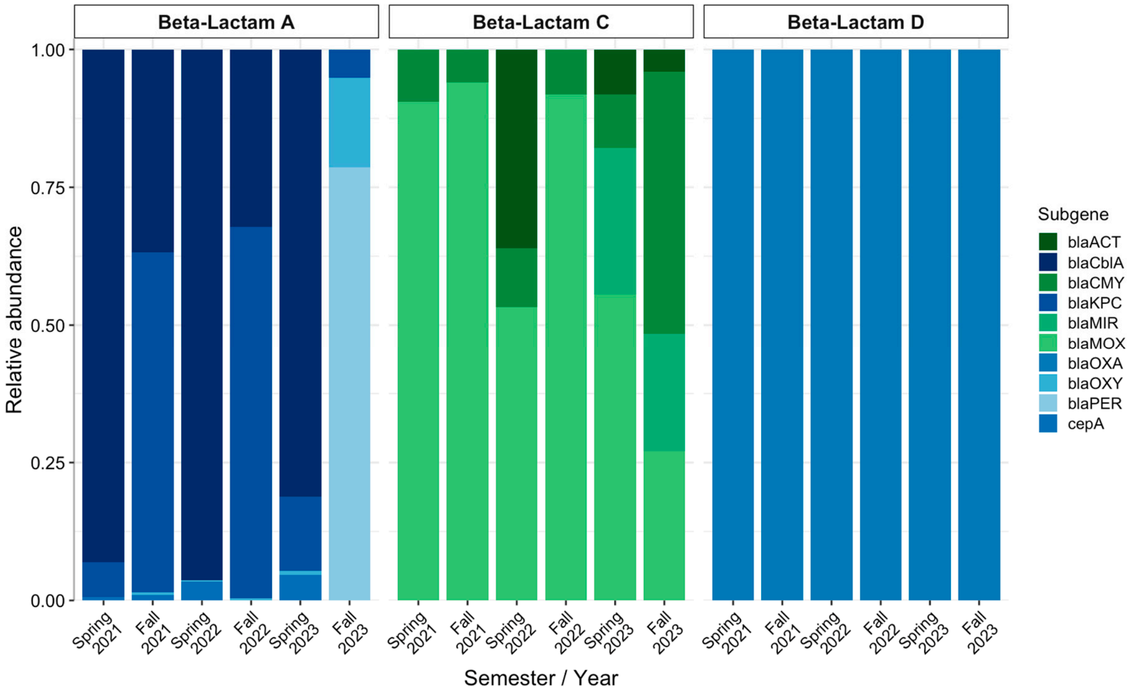

Figure S3. Relative contribution of β-lactam subgenes within enzyme classes (A, C, and D) across Spring and Fall semesters (2021–2023).

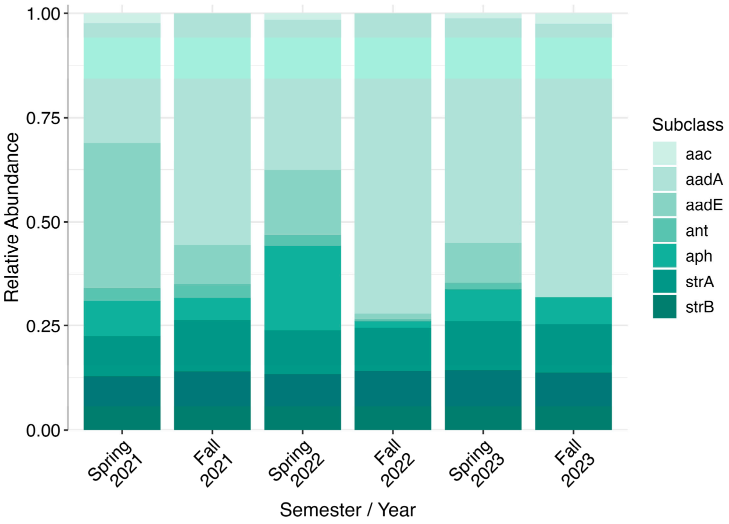

Figure S4. Relative contribution of seven aminoglycoside subclass compositions by semester/year

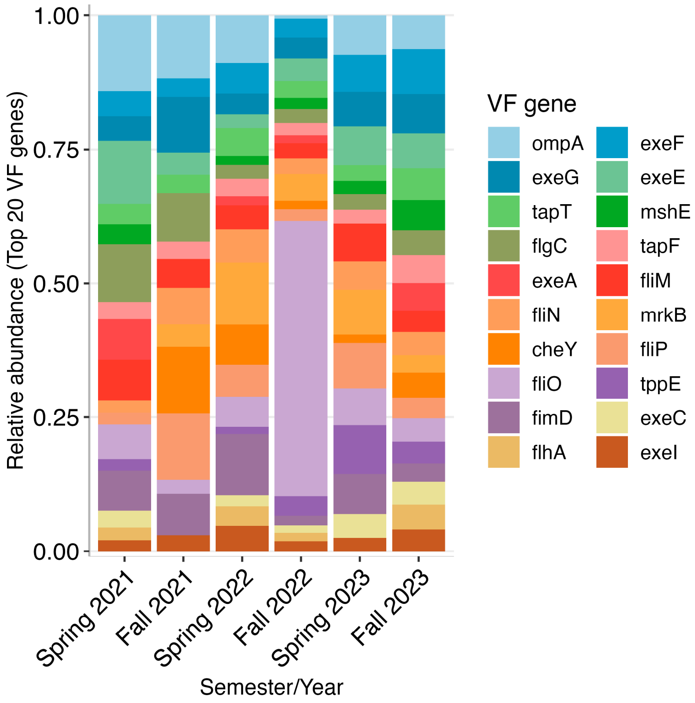

Figure S5. Relative abundance of the top 20 virulence factor (VF) genes across semester/year groups (2021–2023).

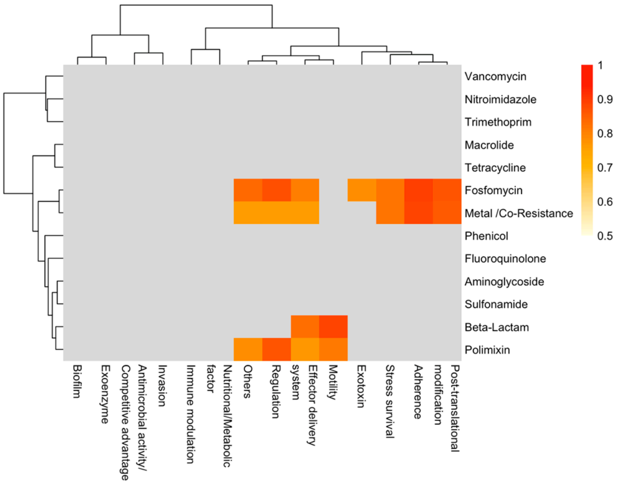

Figure S6. Significant correlations between ARG classes and VF functional categories. Spearman correlation coefficients were calculated using log1p-transformed normalized abundance profiles across wastewater samples. Only statistically significant associations (p-adjusted ≤ 0.05, Benjamini-Hochberg correction) are shown; non-significant pairs are displayed in grey. All surviving associations were positive and strong in magnitude (ϱ = 0.755–0.897). Color intensity reflects Spearman ϱ values ranging from 0.5 (light yellow) to 1.0 (red). Hierarchical clustering was applied to ARG classes and VF categories based on the full correlation matrix to identify shared association patterns.

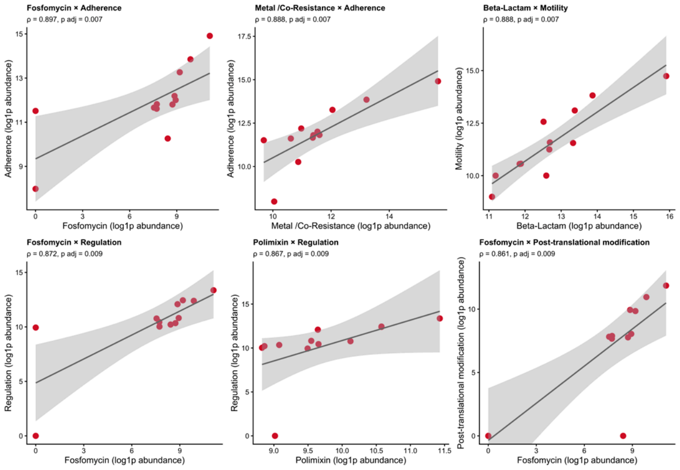

Figure S7. Scatter plots of the six strongest significant ARG classes and VF functional category correlations. Each point represents one wastewater sample (n = 12). Abundance values were log1p-transformed prior to analysis. Lines represent linear fits with 95% confidence intervals. Spearman's ϱ and Benjamini-Hochberg-adjusted p-values are shown for each pair. Two samples with zero fosfomycin abundance (log1p = 0) represent sampling events where fosfomycin resistance genes were not detected.

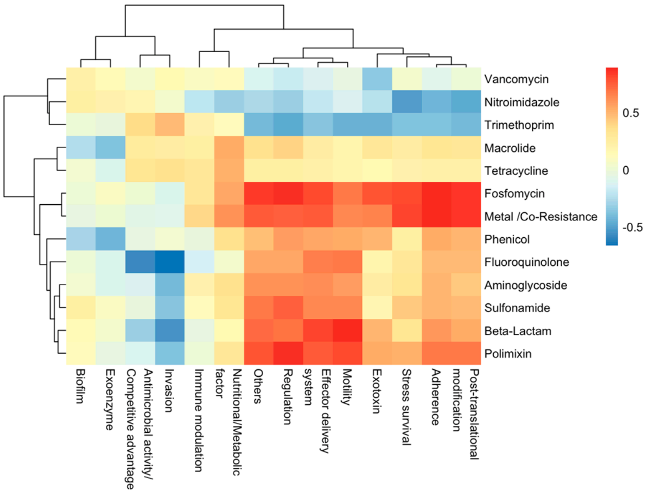

Figure S8. Full correlation matrix between ARG classes and VF categories. Spearman correlation coefficients were calculated using normalized abundance data across samples. Both significant and non-significant associations are shown to illustrate the overall structure of ARG–VF co-variation.

Table S2. Spearman correlation coefficients (ϱ) with bootstrap 95% confidence intervals for the ARG class–VF category pairs significant after Benjamini–Hochberg correction.

**ARG class**	**VF category**	**ϱ**	**95% CI**	**Adjusted p (BH-FDR)**
Fosfomycin	Adherence	0.897	0.542–0.995	0.007
Metal/Co-Resistance	Adherence	0.888	0.597–1.000	0.007
Beta-Lactam	Motility	0.888	0.522–1.000	0.007
Fosfomycin	Regulation	0.872	0.464–0.991	0.009
Polymyxin	Regulation	0.867	0.550–0.993	0.009
Fosfomycin	Post-translational modification	0.861	0.498–0.995	0.009
Metal/Co-Resistance	Post-translational modification	0.859	0.439–0.993	0.009
Fosfomycin	Others	0.837	0.434–0.973	0.016
Beta-Lactam	Effector delivery system	0.825	0.400–0.993	0.018
Metal/Co-Resistance	Stress survival	0.824	0.350–0.982	0.018
Fosfomycin	Stress survival	0.818	0.454–0.978	0.019
Polymyxin	Motility	0.811	0.367–0.964	0.020
Fosfomycin	Effector delivery system	0.809	0.293–0.995	0.020
Fosfomycin	Exotoxin	0.783	0.273–0.994	0.032
Polymyxin	Others	0.783	0.365–0.957	0.032
Polymyxin	Effector delivery system	0.769	0.314–0.943	0.039
Metal/Co-Resistance	Effector delivery system	0.762	0.223–0.978	0.040
Metal/Co-Resistance	Others	0.762	0.241–0.956	0.040
Metal/Co-Resistance	Regulation	0.755	0.208–0.957	0.043

Spearman correlation coefficients (ϱ) with bootstrap 95% confidence intervals (5,000 resamples) are shown for the 19 ARG class × VF category pairs that remained significant after Benjamini–Hochberg correction (adjusted $p \leq 0.05$). Correlations were computed on log1p-transformed relative-abundance profiles across the twelve wastewater samples ($n = 12$). All lower confidence bounds exclude zero. Wide confidence intervals reflect the limited sample size.

Table S3. Collection dates of the twelve wastewater samples selected for shotgun metagenomic sequencing by academic semester and year.

**Year/Semester**	**2021**	**2022**	**2023**
**Spring**	03-23-21<br>04-13-21	01-06-22<br>01-11-22	01-29-23<br>04-17-23
**Fall**	08-13-21<br>08-25-21	08-24-22<br>11-28-22	10-20-23*<br>11-30-23*

*SARS-CoV-2 RT-qPCR negative, all other samples were SARS-CoV-2-RT-qPCR positive [43].

Table S4. Independent open-source taxonomic validation of the Kepler/GenBook classification.

**Sample**	**Semester/Year**	*Acinetobacter* **(%)**	**Dominant genera (Kraken2)**	**Concordant**
10-20-23	Fall 2023	37.0	*Acinetobacter, Citrobacter, Aeromonas*	Yes
11-30-23	Fall 2023	17.8	*Pseudomonas, Aeromonas, Acinetobacter*	Yes
01-29-23	Spring 2023	2.0	*Bacteroides, Phocaeicola, Faecalibacterium*	Yes
08-13-21	Fall 2021	0.4	*Faecalibacterium, Bacteroides, Phocaeicola*	Yes

Table S5. Independent open-source ARG and VF validation for both Fall 2023 samples.

Sample	ARG hits	Key β-lactam / AmpC genes	Other ARG classes	VF hits	Top VF categories

10-20-23	64	*blaCMY*, *bla*ACT, *bla*FOX, *bla*OXA (including carbapenem-active *OXA*-427), *cphA*	fosfomycin, 3× *mcr*, tetracycline, sulfonamide, aminoglycoside, macrolide, fluoroquinolone	898	Motility, Effector delivery, Adherence, Nutritional/Metabolic
11-30-23	36	*bla*CMY-48, *bla*MOX-4, *bla*FOX-7, *bla*PER-3, *bla*OXA	*fosA*, *mcr*-3.17, tetracycline, sulfonamide, aminoglycoside, macrolide, fluoroquinolone	488	Motility, Adherence, Effector delivery, Nutritional/Metabolic

Note: ARG and VF hits represent gene-level ABRicate detections from assembled contigs and indicate gene presence rather than relative abundance.
